# Genetic characterization and curation of diploid A-genome wheat species

**DOI:** 10.1093/plphys/kiac006

**Published:** 2022-02-03

**Authors:** Laxman Adhikari, John Raupp, Shuangye Wu, Duane Wilson, Byron Evers, Dal-Hoe Koo, Narinder Singh, Bernd Friebe, Jesse Poland

**Affiliations:** Department of Plant Pathology, Kansas State University, Manhattan, Kansas 66502, USA; Wheat Genetic Resource Center (WGRC), Kansas State University, Manhattan, Kansas 66502, USA; Center for Desert Agriculture, King Abdullah University of Science and Technology, Thuwal 23955, Saudi Arabia; Department of Plant Pathology, Kansas State University, Manhattan, Kansas 66502, USA; Wheat Genetic Resource Center (WGRC), Kansas State University, Manhattan, Kansas 66502, USA; Department of Plant Pathology, Kansas State University, Manhattan, Kansas 66502, USA; Wheat Genetic Resource Center (WGRC), Kansas State University, Manhattan, Kansas 66502, USA; Department of Plant Pathology, Kansas State University, Manhattan, Kansas 66502, USA; Wheat Genetic Resource Center (WGRC), Kansas State University, Manhattan, Kansas 66502, USA; Department of Plant Pathology, Kansas State University, Manhattan, Kansas 66502, USA; Wheat Genetic Resource Center (WGRC), Kansas State University, Manhattan, Kansas 66502, USA; Department of Plant Pathology, Kansas State University, Manhattan, Kansas 66502, USA; Wheat Genetic Resource Center (WGRC), Kansas State University, Manhattan, Kansas 66502, USA; Department of Plant Pathology, Kansas State University, Manhattan, Kansas 66502, USA; Wheat Genetic Resource Center (WGRC), Kansas State University, Manhattan, Kansas 66502, USA; Department of Plant Pathology, Kansas State University, Manhattan, Kansas 66502, USA; Wheat Genetic Resource Center (WGRC), Kansas State University, Manhattan, Kansas 66502, USA; Department of Plant Pathology, Kansas State University, Manhattan, Kansas 66502, USA; Wheat Genetic Resource Center (WGRC), Kansas State University, Manhattan, Kansas 66502, USA; Center for Desert Agriculture, King Abdullah University of Science and Technology, Thuwal 23955, Saudi Arabia

## Abstract

A-genome diploid wheats represent the earliest domesticated and cultivated wheat species in the Fertile Crescent and include the donor of the wheat A sub-genome. The A-genome species encompass the cultivated einkorn (*Triticum monococcum* L. subsp. *monococcum*), wild einkorn (*T*. *monococcum* L. subsp. *aegilopoides* (Link) Thell.), and *Triticum urartu*. We evaluated the collection of 930 accessions in the Wheat Genetics Resource Center (WGRC) using genotyping by sequencing and identified 13,860 curated single-nucleotide polymorphisms. Genomic analysis detected misclassified and genetically identical (>99%) accessions, with most of the identical accessions originating from the same or nearby locations. About 56% (*n* = 520) of the WGRC A-genome species collections were genetically identical, supporting the need for genomic characterization for effective curation and maintenance of these collections. Population structure analysis confirmed the morphology-based classifications of the accessions and reflected the species geographic distributions. We also showed that *T*. *urartu* is the closest A-genome diploid to the A-subgenome in common wheat (*Triticum aestivum* L.) through phylogenetic analysis. Population analysis within the wild einkorn group showed three genetically distinct clusters, which corresponded with wild einkorn races α, β, and γ described previously. The *T*. *monococcum* genome-wide F_ST_ scan identified candidate genomic regions harboring a domestication selection signature at the Non-brittle rachis 1 (*Btr1*) locus on the short arm of chromosome 3A^m^ at ∼70 Mb. We established an A-genome core set (79 accessions) based on allelic diversity, geographical distribution, and available phenotypic data. The individual species core set maintained at least 79% of allelic variants in the A-genome collection and constituted a valuable genetic resource to improve wheat and domesticated einkorn in breeding programs.

## Teaser

Genotyping diploid A-genome relatives of wheat uncovered high genetic diversity and unique evolutionary relationships giving insight to the effective use of this germplasm for wheat improvement.

## Introduction

Wheat wild relatives are an important reservoir of genetic diversity that can be utilized for wheat improvement, particularly for diseases, insect pests, and abiotic stress tolerance (Wulff and Moscou, 2014). Cultivated tetraploid (pasta wheat, *Triticum turgidum*) and hexaploid (bread wheat, *Triticum aestivum* L.) wheat arose through successive whole-genome hybridization between related species in the *Triticeae*. Although polyploidization in wheat enabled broad adaptation and genome plasticity found in polyploids (Comai, 2005), it also created severe genetic bottlenecks within each subgenome (Feldman and Levy, 2012). Likewise, of the three natural races within wild einkorn, only one natural race (β) has been domesticated, thus, genetic diversity in the wild einkorn is expected to be greater than in domesticated einkorn (Pourkheirandish et al., 2018). Some recent findings, however, reported no or low reduction in nucleotide diversity through einkorn domestication, most likely indicating a minimal bottleneck during domestication of cultivated einkorn (Kilian et al., 2007). This was true when diversity comparisons were performed between wild einkorn-specific races (α and β) versus domesticated einkorn. However, when the comparison was made between the domesticated einkorn versus all groups of wild einkorn, the wild einkorn diversity was much higher than found in the cultivated accessions. The value of A-genome species diversity for alleviating the wheat diversity bottleneck has been described (Mondal et al., 2016; Brunazzi et al., 2018). Thus, diversity assessment in germplasm collections of diploid A-genome species is crucial for conservation planning and efficient utilization of germplasm in breeding.

A-genome wheat species (2*n* = 2*x* = 14, AA) are diploid grasses including the wild einkorn (*T*. *monococcum* L. subsp. *aegilopoides* (Link) Thell.), domesticated einkorn (*T*. *monococcum* L. subsp. *monococcum*), and *Triticum* *urartu* (van Slageren, 1994). Molecular and cytological studies have confirmed that *T. urartu*, a related species sharing the same genome as domesticated einkorn, is the A-genome ancestor to cultivated wheat (*T*. *aestivum*) (Dvorak et al., 1988; Dong et al., 2012). In the first polyploidization event that occurred ∼0.5–0.15 million years ago (MYA) (Charmet, 2011), *T*. *urartu* naturally hybridized with a B-genome donor grass, an extant species but close relative of *Aegilop*s *speltoides* Tausch, giving rise to the wild tetraploid wheat *T*. *turgidum* L. subsp. *dicoccoides* (Körn. Ex Asch. & Graebn.) Thell. (AABB, 2*n* = 4*x* = 28) (Nair, 2019). In the next event, the cultivated tetraploid emmer wheat (*T*. *turgidum* L.) naturally hybridized with the D-genome donor species (*Ae*. *Tauschii* Coss) forming hexaploid bread wheat (AABBDD, 2*n* = 6*x* = 42). The A-genome species morphologically resemble cultivated tetraploid and hexaploid wheat more than any other surviving diploid wheat species and are predominant in the Fertile Crescent (Heun et al., 1997). Domestication of einkorn wheat, together with emmer wheat and barley around 12,000 years ago, transformed human culture from hunting–gathering to agriculture, popularly known as the “Neolithic Revolution” (Kilian et al., 2010). The Karacadağ mountain in the southeast Turkey has been considered the geographical point for einkorn domestication (Brandolini et al., 2016).

The donor of the A genome of the bread wheat, *T*. *urartu*, is estimated to have diverged nearly 0.57–0.76 MYA from another widespread A-genome diploid species, *T*. *monococcum*. Interspecific crosses between *T. urartu* and *T. monococcum* are infertile, confirming the large phylogenetic distance and genetic differentiation of the species (Middleton et al., 2014). Like hexaploid wheat, A-genome species have a large genome size with a mean nuclear DNA content of 5.784 pg/1C in *T*. *urartu* to 6.247 pg/1C in *T*. *monococcum* subps. *aegilopoides* (Özkan et al., 2010). Morphologically, *T*. *urartu* possesses smooth leaves, a brittle rachis, and smaller anthers (<0.3 mm). The wild einkorn (*T*. *monococcum* subsp. *aegilopoides*) are characterized with a brittle rachis, hairy leaves, and larger (≥0.5 mm) anthers. Domesticated einkorn has a non-brittle (semi tough) rachis with smooth leaves (Brandolini and Heun, 2019).

Being homologous to the wheat A-genome, these species provide useful sources for wheat improvement using wide crosses and cytogenetics approaches. The A-genome species are important genetic resources for pest resistance and stress tolerance. For example, *T*. *urartu* was identified as a source of resistance to the root lesion nematode *Pratylenchus thornei* (Sheedy et al., 2012) and stem rust (Rouse and Jin, 2011). Novel stem rust resistance genes *SrTm5* and *Sr60* were mapped in an F_2_ population derived from crosses between wild and the cultivated einkorn (Chen et al., 2018). *Sr35*, the first gene cloned against the devastating stem rust race UG99, also originates from *T*. *monococcum* (Saintenac et al., 2013). A leaf rust gene, *Lr63*, in wheat chromosome 3AS was introgressed from *T*. *monococcum* (Kolmer et al., 2010). Surveying the genetic variation in A-genome species that can be utilized in wheat improvement has lagged, considering the potential value of more effectively utilizing these species for wheat improvement.

Einkorn has multiple botanical names in the literature as proposed by the various taxonomists, and confusion related to the einkorn nomenclature is widespread. In 1948, Schiemann classified einkorn as wild einkorn (*Triticum* *boeoticum* subsp. *thaoudar*), the feral einkorn (*T*. *boeoticum* subsp. *aegilopoides*), and the domesticated einkorn (*T*. *monococcum* subsp. *monococcum*) (Schiemann, 1948; Brandolini et al., 2016). Mac Key published einkorn classification in 1954 (Mac Key, 1954) and updated the nomenclature several times through 2005 (Mac Key, 2005a). van Slageren also published the einkorn nomenclature, where the wild and domesticated einkorn was simply named as *T*. *monococcum* L. subsp. *aegilopoides* (hereafter subsp. *aegilopoides*) and *T*. *monococcum* L. subsp. *monococcum* (hereafter subsp. *monococcum*), respectively (van Slageren, 1994). In this study, we follow van Slageren (1994) einkorn taxonomy, because the A-genome species collection in the Wheat Genetics Resource Center (WGRC) at Kansas State University (KSU) was initially classified using this nomenclature (van Slageren, 1994).

A well-characterized population structure of A-genome species is critical to formulating effective conservation strategy, selecting diverse germplasm, and enhancing the accuracy of the genomic analysis with structure information (Singh et al., 2019). Population structure and diversity assessment have become easier with next-generation sequencing, which makes discovery of thousands of genotyping markers possible. Here, we used genotyping by sequencing (GBS) for single nucleotide polymorphism (SNP) discovery. GBS is straightforward, high-throughput, and with multiple downstream pipelines for data processing (Poland et al., 2012a). However, population structure of A-genome species has not been evaluated in detail with the resource of whole-genome profiling. Therefore, our objectives are to: 1) curate A-genome wheat accessions in the gene bank by recognizing genetically identical and misclassified accessions, 2) assess the population structure and genetic diversity of the A-genome wheat species, and 3) establish genetically, geographically, and phenotypically representative core collections for A-genome species within the WGRC gene bank.

## Results

### A-genome species distribution

**Figure 1:**
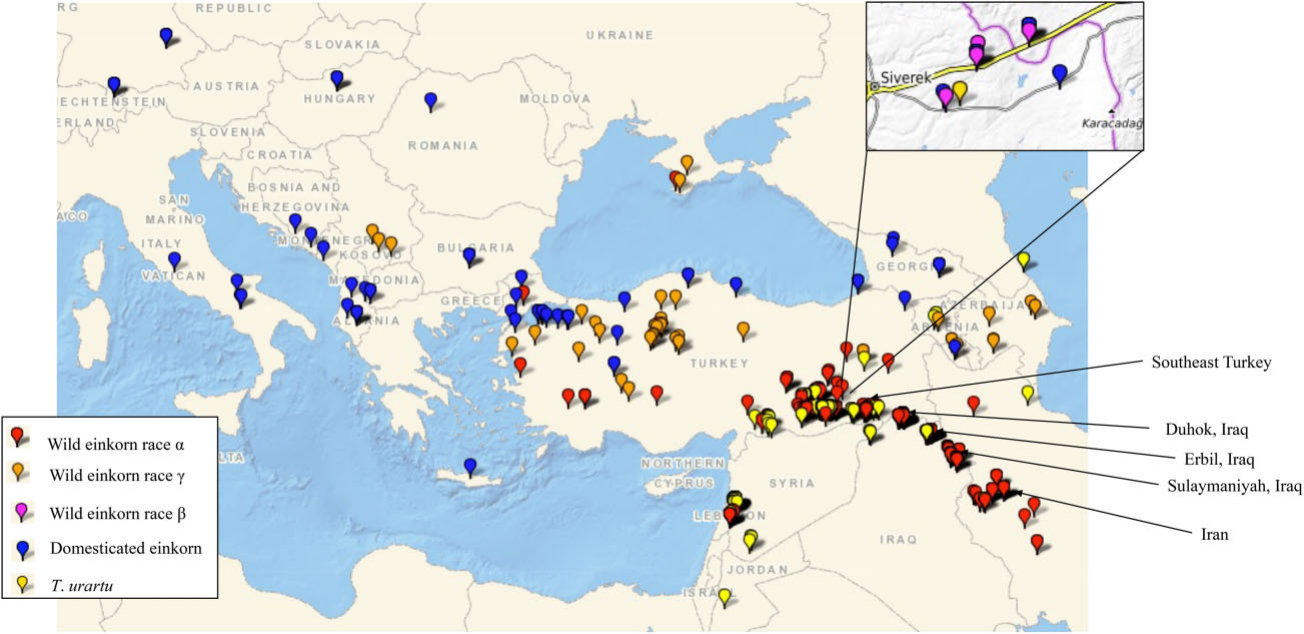
Geographic distribution of A-genome wheat species maintained in the WGRC gene bank. Collection sites of accessions in this study are designated for domesticated einkorn (*T*. *monococcum* subsp. *monococcum*) (blue); α race within wild einkorn (*T*. *monococcum*. subsp. *aegilopoides*) (red); γ race wild einkorn (orange); β race wild einkorn (magenta); and *T*. *urartu* (yellow).

Most of the wild einkorn (subsp. *aegilopoides*) in our collection, were collected across Turkey, northern Iraq, west Iran, and Transcaucasia, whereas the majority of domesticated einkorn (subsp. *monococcum*) were from west Turkey and the Balkans (Figure 1 and Supplemental Table S1). About half of the *T*. *urartu* accessions were from eastern Lebanon, around the Beqaa Valley, and a major part were from southeast Turkey (Figure 1). The A-genome species are known to span from Transcaucasia through Anatolia to the Caspian Sea. Thus, the WGRC collection generally covers the geographic range of this species. After genomic characterization including misclassified accessions adjustment, we retained 196 *T*. *urartu* accessions, 145 domesticated einkorn accessions, and 584 wild einkorn accessions (Supplemental Table S1). There were also five tetraploids identified in the population which were curated to correct species designations.

### Markers and genotyping

For all A-genome accessions, we identified 44,215 biallelic SNPs after a filter for passing Fisher exact test of disassociated alleles (doi:10.5061/dryad.9zw3r22f6). Upon filtration (minor allele frequency [MAF] > 0.01, missing <30%, heterozygosity <10%) and species separation, we retained 13,860 SNPs in total comprising 3,840 SNPs for *T*. *urartu*, 8,989 SNPs for *T*. *monococcum*, 8283 SNPs for subsp. *aegilopoides*, and 4,401 for subsp. *monococcum* (Table 1). We observed loci that were fixed or otherwise a single heterozygous genotype call within the individual species and subspecies. To compute total segregating loci per group and minimize the effect of potential sequencing error, we further filtrated and removed any loci that had only a single accession with heterozygous genotype and were otherwise fixed in remaining population, if the population size was >100. For A-genome species diversity assessment, thousands of segregating loci were available for the groups defined by population analysis and core set selections (Table 1). For wheat and A-genome diploids together we found 15,300 filtered SNPs and used the information to see specific relationships between the species.

**Table 1:** A-genome species and sub-species groups with number of samples, the Nei’s diversity indices, and number of segregating loci

Group	Number of samples	Diversity index	Segregating SNPs (%)
A-genome species (*T. monococcum* + *T. urartu*)	925	0.25	13,860 (100)
* T*. *monococcum* (einkorn)	729	0.106	8,989 (64.8)
* *Domesticated einkorn (subsp. *monococcum*)	145	0.062	4,401 (31.7)
* *Wild einkorn (subsp. *aegilopoides*)	584	0.087	8,283 (59.7)
* *α race einkorn	524	0.075	7,159 (51.6)
* *γ race einkorn	48	0.095	5,422 (39.2)
* *β race einkorn	12	0.065	3,402 (24.5)
* T*. *urartu*	196	0.066	3,840 (27.7)
A-genome species core set	79	0.261	13,654 (98.5)
* T*. *monococcum* core	60	0.117	7,416 (82.5)
* *Domesticated einkorn core	19	0.068	3,765 (85.5)
* *Wild einkorn core	41	0.099	6,576 (79.4)
* T*. *urartu* core	19	0.066	3,286 (85.5)

The percentage of segregating SNPs for core set groups were estimated relative to the segregating loci within the respective groups.

### Gene bank curation

We identified and corrected a total of 22 misclassified accessions using fastStructure analysis, phylogenetic (Supplemental Figure S1) and PCA clustering including nine *T*. *urartu*, two subsp. *monococcum*, six subsp. *aegilopoides*, and five tetraploid accessions. As a large number of accessions in both *T*. *urartu* and subsp. *aegilopoides* were from southeast Turkey, we observed most of the misclassified accessions also were from the same site (Supplemental Tables S1 and S2).

While evaluating the collection for genetically identical accessions, we compared various number of loci for allele matching per A-genome species (Table 2) as the SNPs were filtered to keep only the sites with MAF > 0.05, ˂50% missing, and ˂10% heterozygous. We identified and used a threshold of ≥99% identity by state to declare the individuals as genetically identical accessions (Supplemental Figure S2) with tolerance for sequencing and genotyping error. With these criteria we identified a total of 520 (56%) accessions within the collection having at least one other accession that was genetically identical. These identical accessions were mostly observed within *T*. *urartu* (135) and within α race subsp. *aegilopoides* (326) (Table 2 and  Supplemental Table S1). To confirm this analysis, we checked the collection sites of the groups of genetically identical accessions and identified that all of the respective matching accessions were collected from the same or nearby sites. We further observed the identical accessions had matching phenotypes such as glume color, for which the scores were the same for all accessions within the respective sets of identical accessions (Supplemental Table S1). For instance, accession no. TA471 had 11 other identical accessions which all had glume color score of 7 on a scale of 1 (white) to 9 (black) (Supplemental Table S1). This further confirmed the utility and accuracy of using the GBS data for identification of genetically identical accessions.

**Table 2:** Number (#) of unique accessions, number of accessions in a set consisting maximum identical accessions, and total accessions of A-genome species: *T*. *urartu*, domesticated einkorn (subsp. *monococcum*), and wild einkorn (subsp. *aegilopoides*) three genetic races: α, γ, and β

	α Race	γ Race	β Race	subsp. *monococcum*	*T*. *urartu*
Total accessions	524	48	12	145	196
# Loci compared	4,112	4,112	4,112	3,337	6,356
Identical^a^	28	3	0	5	39
Unique accessions (%)	198 (37.8)	37 (77)	12 (100)	97 (66.8)	61 (31.2)

The identical accessions were detected using pairwise allele matching.

^a^ Number of accessions in a set that represent the largest group of genetically identical accessions.

### Relationship between A-genome diploid and wheat

The genetic grouping of A-genome diploids and CIMMYT wheat lines together showed that wheat is closer to *T*. *urartu* than to *T*. *monococcum* (Supplemental Figure S3), a finding in agreement with the known relationship between the species. The unrooted neighbor-joining (NJ) tree constructed for wheat and A-genome diploid wheat showed five accessions (TA282, TA10915, TA1325, TA1369, and TA10881) clustering far from the *T*. *urartu* major clade (Supplemental Figure S3). Cytological analysis identified them as tetraploid (2*n* = 28) (Supplemental Figure S4). Therefore, we corrected the species designations for these five accessions and excluded them from further population analysis. This observation confirms that GBS also enables identifying cryptic accessions with different ploidy levels in the population.

### A-genome population structure and wild einkorn genetic races

**Figure 2:**
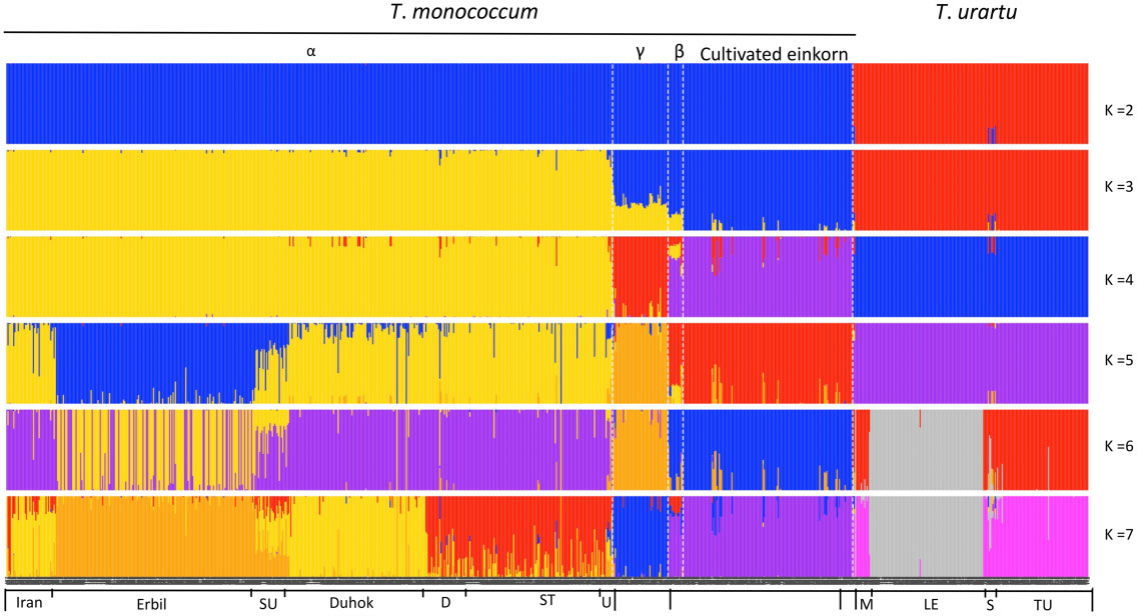
Population structure of A-genome wheat species (*T. monococcum* L. and *T*. *urartu*), where description of the groups and colors refers to *K* = 7. Subpopulations were determined using fastStructure. Each color represents a population and each bar indicates the admixture proportion of an individual accession from K populations. The subgroup within α, which is exemplified by orange color includes the accessions from Erbil (also spelled Arbil), Iraq, whereas the subgroup embodied by yellow only comprises the accessions from Duhok (ancient name “Dahuk,” Iraq). The bars with mostly red color represent the accessions from southeast Turkey (ST). Other admixture types within α included accessions were from Iran, SU (Sulaymaniyah [Iraq]), random different sites (D) and unknown sites (U) as indicated. Within *T*. *urartu*, the LE group represents accessions from Lebanon, the TU includes accession from Turkey, S indicates accessions from Syria, and M shows accessions from mixed sites.

Population grouping in the fastStructure analysis at *K* = 2–7 showed the A-genome genetic structure was split with the known biological and geographical characterizations (Figure 2). This analysis revealed a number of misclassified accessions that were individually curated and checked, including morphological confirmation, and were reclassified to the appropriate group (Supplemental Table S2).

At *K* = 2, the population differentiation occurred only at the level of species, the accessions split into *T*. *monococcum* and *T*. *urartu*, confirming known species differences (Figure 2). At *K* = 3, the two subspecies of *T*. *monococcum* differentiated with the accessions in the α wild einkorn race were clearly differentiated from domesticated einkorn. However, the other races of wild einkorn (β and γ) appeared to be an admixture, supporting that there is incomplete differentiation between the wild and domesticated einkorn, a classification that is simply based on the few morphological characteristics of the domestication syndrome.

We observed differentiation of wild einkorn into genetically distinct subgroups at *K* = 4. Comparing these three wild einkorn subgroups with the α, β, and γ wild einkorn races described by Kilian et al. (2007), we reported the three genetic subgroups as representing the races α, β, and γ by identifying common USDA Plant Introduction (PI) numbers for accessions in both studies. The genetic clustering pattern and geographical distribution then confirmed that the subgroups within subsp. *aegilopoides* represents α, β, and γ races described and we hereby name these genetic groups accordingly (Supplemental Table S3; Kilian et al., 2007). In Kilian et al. (2007), the α race accessions were primarily from southeast Turkey, northern Iraq, and Iran; the γ race involves accessions from Transcaucasia to western Anatolia; and the β race comprises a few accessions collected around Karacadag Turkey (Figure 1 and  Supplemental Table S1). Based on population differentiation, α race exhibited the strongest differentiation with domesticated einkorn and should represent the base population of subsp. *aegilopoides*, whereas the β race of wild einkorn exhibited the least differentiation with subsp. *monococcum*. Interestingly, the β race did not fully differentiate from subsp. *monococcum* at any number of K (Figure 2), (1) supporting that domesticated einkorn originated out of this subpopulation, which already largely differentiated from the other wild einkorn, or (2) that the β race represents “feral” subsp. *monococcum* accessions that were, at one point, fully domesticated but reverted to wild plant types through introgression and admixture.

At *K* = 5, the population subgrouping according to the accession origin was observed in α race accessions within the wild einkorn. Accessions from Erbil (ancient name “Arbil”) differentiated as a subpopulation, and the accessions from Sulaymaniyah (Iraq) split as the admixture of the Erbil subgroup and the remaining accessions at *K* = 5 (Figure 2). We could not observe any new differentiation within the wild einkorn group at *K* = 6. However, at *K* = 7, we observed three distinct subgroups and a higher level of admixture within the α race of subsp. *aegilopoides* (Figure 2). Also, there were two main sets of admixture types; the first set mainly consists of accessions from Iran that shared ancestry from the Duhok and Turkey subgroups, and the second corresponds with accessions from Sulaymaniyah (Iraq) and has ancestry from all three subgroups. Hence, within the population of α race einkorn accessions, three subgroups exist; Erbil, Duhok, and southeast Turkey, and two groups of genetic admixtures (Iran and Sulaymaniyah), named from their origin.

We did not observe any subgrouping within the accessions from the southeast Turkey, yet the accessions were primarily from two sites (Sanliurfa and Mardin). The grouping pattern of three subgroups within the α race accessions provided an insight into the wild einkorn subgrouping and their genetic relationships. We did not observe within population differentiation in domesticated einkorn group.

In *T*. *urartu*, the subgrouping occurred at *K* = 6, and was unchanged at *K* = 7 (Figure 2). Two major *T*. *urartu* subgroups represented accessions from Turkey (TU) and another from Lebanon (LE). Few *T*. *urartu* accessions were from Syria (S); some showed admixture, and some had a clean ancestry that resembled accessions from Turkey (Figure 2). The few remaining accessions primarily were from Transcaucasia and exhibited an ancestry similar to accessions from Turkey (Figure 2).

### Phylogenetic clustering and PCA

**Figure 3:**
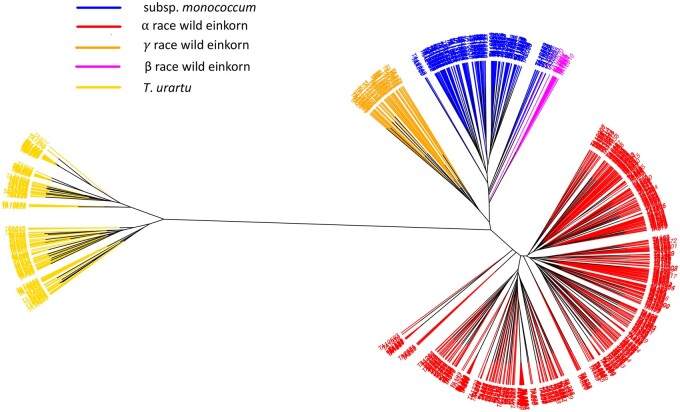
An unrooted NJ tree of A-genome species: *T*. *urartu*, subsp. *aegilopoides*, and subsp. *monococcum*. The tree branches are colored based on the genetic grouping of the accessions after correcting misclassified accessions. *T*. *urartu* (yellow), domesticated einkorn (blue), wild einkorn race α (red), wild einkorn race γ (orange), and wild einkorn race β (magenta) are shown.

The phylogenetic clustering split the A-genome accessions into separate clades for *T*. *urartu*, *T. monococcum* subsp. *monococcum*, and all races within the subsp. *aegilopoides* (Figure 3). Only 12 accessions were retained within race β, and the accessions were clustered with some other domesticated einkorn accessions (Figure 3). The *T*. *urartu* clade distantly clustered in phylogenetic analysis from either of the einkorn clade indicating the obvious genetic differences between species. The misclassified accessions (Supplemental Figure S1) observed in the phylogenetic clustering were re-classified into proper genotype-based classes.

A principal component analysis (PCA) plot of A-genome species also showed accessions clustering as in fastStructure and phylogenetic analysis (Supplemental Figure S5). The first principal component (PC1), which grouped the accessions of *T*. *monococcum* and *T*. *urartu* in two primary clusters, explained 58% of the variation. The PC2, which divided the einkorn accessions, explained 8% of the variation and separated domesticated and different races within the wild einkorn. Misclassified accessions previously observed also were revealed in the PCA analysis and their taxonomy classification adjusted.

### Genetic diversity and F_ST_

A considerably high Nei’s diversity index (0.25) was observed for the complete set of A-genome accessions. The Nei’s diversity indices for individual A-genome species ranged from 0.062 for domesticated einkorn to 0.106 for the entire einkorn group. Among the three races of wild einkorn, the Nei’s diversity indices of β race (0.065) was the lowest and γ was the highest (0.095; Table 1). As expected for diverse accessions, we found a high density of alleles with low MAF (Supplemental Figure S6).

Population differentiation within the A-genome species was further verified by pairwise fixation index (F_ST_) values (Nei, 1987) computed between the groups. Pairwise F_ST_ between *T*. *urartu* and entire einkorn was >0.80, supporting that the two species are strongly differentiated (Table 3). The pairwise F_ST_ (0.56) between the α race and domesticated einkorn indicated the strongest differentiation between any two groups within the einkorn, whereas the weakest differentiation (F_ST_ = 0.31) was between the β race and domesticated einkorn, supporting the model that this wild race was the most likely forerunner of domesticated einkorn as previously hypothesized (Kilian et al., 2007). The concept also was endorsed by the origin of β race einkorn in the WGRC collection, mostly from Diyarbakir and Sanliurfa, which are near Karacadag and Kartal-Karacadag mountains—the likely points of domestication. Nonetheless, the genetic grouping of β also occurred with subsp. *monococcum* in the unrooted NJ tree (Figure 3). Pairwise F_ST_ (∼0.40) between pairs: “γ race - subsp. *monococcum*” and “γ race - α race” implicit the differentiation of γ race as a genetically intermediate type from truly wild α race and domesticated einkorn (Table 3). The pairwise F_ST_ computed between two subpopulations (Turkey and Lebanon) of *T*. *urartu* was 0.52, which also agrees with the population structure analysis.

**Table 3:** Pairwise F_ST_ coefficients among the A-genome wheat species

	α Race	γ Race	β Race	*T*. *urartu*
subsp. *monococcum*	0.56	0.41	0.31	0.87
α race	–	0.40	0.50	0.86
γ race	–	–	0.37	0.83
β race	–	–	–	0.86

Higher F_ST_ reflects a stronger population differentiation. The α, β, and γ genetic races comprise the wild einkorn (*T*. *monococcum* subsp. *aegilopoides* L.).

Pairwise F_ST_ computed between the subpopulations within α race of subsp. *aegilopoides* signaled the geographical differentiation and the potential gene flow within this wild einkorn race. Consistent with the fastStructure output, the Erbil subgroup showed the stronger differentiation (higher F_ST_) with other wild einkorn subgroups (Supplemental Table S5). The subgroup Duhok and southeast Turkey and their admixture group (Iran) had the minimum pairwise F_ST_ (∼0.12). The accessions within the admixture group of Sulaymaniyah displayed almost similar differentiation (∼F_ST_ = 0.16) with three subgroups, which agrees with population structure as the admixture group has ancestry from all three.

### F_ST_ scan and einkorn selection signature

After filtration and imputation, we had 6,622 SNPs segregating in *T*. *monococcum* on which we calculated per site F_ST_ values for each of the seven chromosomes that ranged from near 0 to 1. Both methods, Porto-Neto et al. (2013) and VCFtools, produced similar results for raw and smoothed F_ST_ values. We used a genome-wide threshold of 3σ (0.24) over the mean F_ST_, from which we observed only a single-selection signature on short arm of chromosome 3A (Supplemental Figure S7) after smoothing using Lowess method (*f* = 0.1) (Pintus et al., 2014). This selection signature corresponded to the locus that harbors the Non-brittle rachis 1 (*Btr1*) (Pourkheirandish et al., 2018) and was supported by the BLAST hit of a coding sequence (Supplemental Text S1) of *Btr1* on the reference genome used to genotype our population (*T*. *urartu* pseudomolecule), which was occurred at 62 Mb on chromosome 3A. We also observed that the raw F_ST_ values for three consecutive sites of the region (62 Mb) had the highest (F_ST_ = 1) values. Thus, this selection scan identified the impact of strong selection for *Btr1* in the domesticated einkorn.

### A-genome core collection

**Figure 4:**
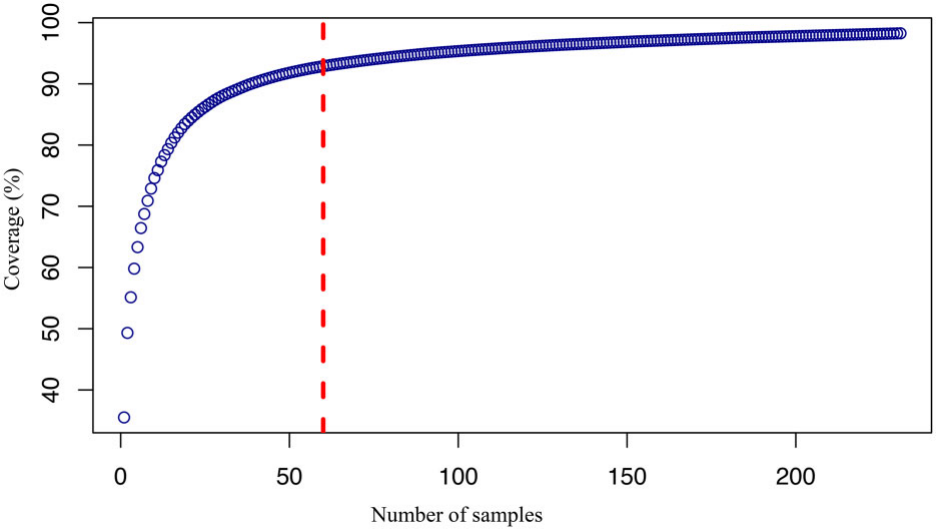
Relationship between the allele coverage as estimated using GenoCore and the number of samples selected in the core for einkorn group (*T*. *monococcum*). The threshold for 60 accessions at ∼90% genotype coverage is shown with vertical red line.

To maximize the utility of the WGRC collection we identified a core set that captured the majority of total allelic diversity within 19 *T*. *urartu* accessions, and 60 accessions of *T*. *monococcum* (Supplemental Table S4). In core sets of the entire A-genome collection, we captured ∼98% of the identified alleles (Figure 4), whereas each separate sub-core also captured at least 79% of the segregating alleles of the respective species-specific collections (Table 1). Enriching allelic diversity within the smaller core collections was confirmed by the higher Nei’s diversity index (0.26) of the selected core sets relative to the entire collection (0.25) (Table 1). Distribution of the core set accessions in the phylogenetic cluster, PCA clusters, and in the geographic map showed that the selected accessions also represented all subgroups within the population and covered the geographic range (Supplemental Figures S8–S10). Ranges of glume color scores (Supplemental Table S4) in the core indicated that the core collections are also an excellent representative of phenotypic variations within the whole collection.

## Discussion

### A-genome species distribution, einkorn nomenclatures, and morphology

Our results confirm that the WGRC A-genome collection includes arrays of naturally selected germplasm around the center of origin (Figure 1). While verifying the morphologically based grouping of A-genome species through population analysis, we identified three genetically different wild einkorn races (Figures 2 and 3). This information is very crucial in handling a large group of wild einkorn so that accessions with desired genetic background and morphology of interest can be selected for utilization in breeding and further investigation. The wild einkorn genetic races described herein, matched with the races described in Kilian et al. (2007), add information to establish the evolutionary and genetic relationships between wild and domesticated einkorn wheat. However, various nomenclature of the einkorn (Supplemental Figure S11) creates a conundrum in interpreting the different races within the wild einkorn. Some einkorn nomenclature is written in multiple languages; Schiemann (1948) published her nomenclature in German and in Russian (Dorofeev et al., 1979), which could have reduced the acceptance of the nomenclatures by the wider research communities. In a revised form of Mac Key’s classification (Mac Key, 2005b), the *T*. *monococcum* subsp. *boeoticum* was changed to *T*. *monococcum* subsp. *aegilopoides* (Goncharov, 2011). Therefore, no single einkorn classification is deemed to be the most widely accepted and uniformly used. The van Slageren (1994) nomenclature that we follow also is mostly in agreement with the Mac Key classification, because both systems use *T*. *monococcum* subsp. *aegilopoides* as the wild einkorn. With all these issues, an updated and widely accepted monograph of einkorn may help maintaining uniformity in taxonomy of these A-genome species.

Species and subspecies classification is first based on morphology. Multiple studies also have discussed different ecogeographical wild einkorn races that have intermediate morphology. van Zeist (1992) described two groups of wild einkorn: the first group (*T*. *boeoticum* subsp. *thaoudar*) predominately exists in the southeast Turkey, northern Iraq, and west Iran, and the second group (*T*. *boeoticum* subsp. *aegilopoides*) primarily occurs in the west Anatolian center (van Zeist, 1992). The first group of accessions had a double-grained spikelet, and the second group was single-grained, suggesting that the second group is more similar to domesticated einkorn. Brandolini and Heun (2019) explained the *T*. *boeoticum* subsp. *aegilopoides* as an intermediate type feral (semi-wild) einkorn with a semi-brittle rachis and *T*. *boeoticum* subsp. *thaoudar* as the truly wild einkorn with an extremely brittle rachis and argued on the quantitative nature of brittleness in einkorn wheat. They hypothesized that the feral einkorn had evolved when agriculture moved from the southeast to western Turkey and the Balkans. The semi-brittle rachis breaks into two parts only after being bent and the naturally emerged semi-brittle rachis einkorn mutant still exists in the vicinity of the Karacadag, however, the area is predominant for the truly wild double-grained einkorn (Brandolini and Heun, 2019). Some einkorn accessions with intermediate leaf hairiness, a trait used to classify accessions that is common only in wild einkorn, was also observed (Empilli et al., 2000), indicating that einkorn with intermediate or intergraded morphological characteristics are common (Harlan and Zohary, 1966). The three genetic races of wild einkorn observed in this study also possess unique genetic relationships with cultivated einkorn, as shown by phylogenetic grouping and pairwise F_ST_ values, showing the varying levels of relatedness within and between wild einkorn accessions.

### Gene bank curation

Globally, plant gene banks often suffer from identified and unidentified genetically identical accessions that unnecessarily increase maintenance costs and more acutely can result in duplicated efforts when characterizing and utilizing the germplasm (Díez et al., 2018). Here, we curated 930 A-genome species accessions in WGRC gene bank, identifying the identical and misclassified accessions, and recognizing valuable unique accessions using genotyping. The existence of misclassified accessions in the gene bank may be due to human error on class assignment and/or data recording. It is also possible that some accessions might also have controversial, intermediate or ambiguous morphology. Also, possible seed mixing during germplasm management or germplasm exchanging between gene banks can lead confusion about accessions, particularly as accessions are often renumbered when entered into a different gene bank and original matching accession numbers lost. As an example of misclassification, consider the wild einkorn accession PI 427328. Except for the WGRC and Leibniz gene banks, three other collections have listed this accession (PI 427328) as *T*. *urartu* (https://www.genesys-pgr.org/a/v2JRrMq2g22), illustrating the importance of genetic scrutiny of the misclassified accessions within the A-genome accessions in different repositories.

One caveat is that the analysis was based on a single plant DNA and the variants were from a single seed per accession. While the accessions were purified and derived from single plants, inferences based on these data may not fully represent possible heterogeneity in various accessions. Depending on the origin, maintenance, and propagation of different accessions in different gene banks there may be varying levels of heterogeneity within a given accession. To fully observe the heterogeneity of an accession, even for inbreeding species, testing multiple individuals to represent a given accession is recommended. However, the observations we made in the experiment even with a single plant genotyping provide an excellent picture of overall diversity and roadmap for selecting optimal sets of accession for next step such as for breeding and core set development. While it is not advocated to remove accessions from the gene banks, overall, this genotype-based curation can give needed focus to limited resources. Having a nonredundant accession list, along with core collections, can reduce the gene bank operation costs and make germplasm preservation and utilization easier, particularly anytime the collection needs to be increased or when phenotypic evaluations are undertaken.

### *Triticum urartu*: the closest A-genome diploid relative of wheat

With GBS information here we showed that *T*. *urartu* is the closest diploid A-genome relative of wheat and thereby most likely donor of A-genome to the hexaploid wheat (Supplemental Figure S3). Most previous studies describing the relationship between *T*. *urartu* and wheat (Dvořák et al., 1993), however, relied on cytogenetic analysis. This study agrees with the known relationships between wheat and A-genome diploids while using a much larger set of accession and molecular markers (∼1,000 diploids and >200 wheat) than previous studies.

### Wild einkorn races

The wild einkorn groups were previously divided into α, β, and γ races (Kilian et al., 2007; Zaharieva and Monneveux, 2014) which was consistent with the phylogeny observed in our study. Furthermore, we validated these race groups to match accessions with common USDA PI numbers in both studies overlapped their point of collection and almost all fall under the same races in both studies (Supplemental Table S3). Comparing between studies, there were a few discrepancies in race assignment of accessions (Kilian et al., 2007) that needed correction. For example, Kilian et al. (2007) grouped PI 427328 in *T*. *urartu*, but our genetic analysis grouped it into α race within subsp. *aegilopoides* which is also in harmony with WGRC database (accession no. TA879). According to the Genesys database (https://www.genesys-pgr.org/10.25642/IPK/GBIS/98704), another gene bank (Leibniz Institute of Plant Genetics and Crop Plant Research) also classified this PI 427328 within wild einkorn but under the name *T*. *baeoticum* Boiss. subsp. *boeoticum* exemplifying multiple wild einkorn nomenclatures use and creating confusion when describing wild einkorn. Interestingly, Kilian et al. (2007) reported a few feral types of einkorn accessions, indicating they are *T*. *monococcum subsp*. *aegilopoides* according to the nomenclature used, which we did not observe in the WGRC collection. We showed that the wild and domesticated einkorn can clearly be differentiated based on genomic data into α, β, and γ races and the domesticated accessions. Given the difficulty and ambiguity of morphological classification, the genetic classification from genomic data can be a preferred approach to cleanly classify any given accession.

### Population analysis and different groups under A-genome species

The population structure and F_ST_ analysis on the A-genome species endorsed the established relationships between the species and subspecies. For instance, hybrids between *T*. *monococcum* and *T*. *urartu* are largely sterile and, hence, the genetic differentiation between these species is apparent (Fricano et al., 2014). Also, the intraspecific population differentiation between groups under einkorn at relatively higher K supported the known genetic relationship between these crossable subspecies that produce mostly fertile hybrids (Harlan and Zohary, 1966).

Our analysis shows that the α race einkorn accessions most likely represent the truly wild einkorn with an extremely brittle rachis, most likely the group of accessions that were traditionally classified as *T*. *boeoticum* subsp. *thaoudar* (Brandolini and Heun, 2019). Differentiation of subpopulations within the α race wild einkorn corresponding to geographic distribution implies migration and genetic drift among truly wild einkorn in the Near East. The *T*. *urartu* subgrouping of accessions from Lebanon and Turkey agrees with Wang et al., (2017), where two subgroups, Mediterranean coastal and Mesopotamia–Transcaucasia, within *T*. *urartu* were reported (Wang et al., 2017).

### Diversity analysis

Cultivated einkorn had a lower Nei’s diversity index (0.062) than the wild sister group and wild *T*. *urartu* (Table 1), which was expected. As a domesticated species, subsp. *monococcum* experienced a population bottleneck and artificial selection contributed to further limit genetic diversity. On the other hand, the population structure of cultivated einkorn did not show substantial admixture. With the exception of a few accessions, all individuals were true to single ancestry (Figure 2), suggesting a low post domestication admixture contributing elevated diversity. The involvement of a single race (*β*) in domestication would have further reduced allelic diversity in the cultivated einkorn. Kilian et al. (2007) illustrated no nucleotide diversity was reduced during einkorn domestication; instead, they observed increased diversity in domesticated compared with wild einkorn (Kilian et al., 2007). However, the diversity assessment in Kilian et al. (2007) could be influenced by the limited number of loci and smaller sample size; especially, diversity estimates are sensitive to sample size when there are only a handful of markers (Bashalkhanov et al., 2009; Li et al., 2009). In this experiment, we used thousands of SNP markers and have larger number of accessions, which minimized the effect of sample size and the number of loci. The highest Nei’s diversity index (0.25) for all A-genome combined and the considerably higher Nei’s diversity index for each species and core collections indicated that these accessions are very important assets with novel and useful genetic variations.

### *Btr1*: einkorn domestication signal

Through F_ST_ computation, we showed that in einkorn wheat there is a single strong selection signal observed on chromosome 3A corresponding to the *Btr1* locus (Supplemental Figure S7). Previous study also described *Btr1* as one of the most important features of einkorn domestication (Pourkheirandish et al., 2018). The non-brittleness in domesticated einkorn is controlled by a single nucleotide change in *Btr1* of wild einkorn that results in an amino acid substitution (alanine to threonine) (Pourkheirandish et al., 2018). With ∼1,000 filtered loci per chromosome, we located the candidate selection region. The availability of a *T*. *monococcum* reference genome to call the genotype would be ideal for obtaining dense markers and better locating the selection signature on einkorn wheat.

### Core collections

Establishing core collections of A-genome species enabled the harnessing of useful genetic variation to improve wheat and cultivated einkorn; this genetic core of the A-genome species included only 79 accessions and yet contains ∼98% of the identified alleles while achieving a more than ten-fold (79/930) reduction in the number of accessions (Table 1 and Supplemental Table S4). The higher Nei’s diversity index computed for these core collections supported that they have considerably higher relative diversity and can be leveraged for targeted germplasm improvement.

### Conclusions

This study reports the important aspects of the A-genome wheat species for genetic diversity, gene bank curation, and core set selection. Following an assessment of nearly 1,000 accessions, we reported that the A-genome species possess a considerable amount of genetic diversity, which can be utilized in breeding wheat and domesticated einkorn. This vast diversity is most effectively managed in prebreeding with well-defined core collections. Identifying and in-depth characterizing of such core collections adds substantial value and accessibility to the germplasms. Having a well curated and accurately described gene bank collection, as done here, is a critical foundation to effectively using this rich diversity for crop improvement and enhancing the value of gene bank resources.

## Materials and methods

### Plant resources

This study included 930 accessions of the A-genome diploid wheat species maintained in the WGRC gene bank (Supplemental Table S1), which were primarily acquired from the Near East, Transcaucasia, and the Balkans (Figure 1). Most of the A-genome accessions (∼85%) tested include those initially collected by B. Lennert Johnson, University of California–Riverside in the 1960s and 1970s. The remaining accessions were obtained from gene banks in Japan (22), Germany (24), and ICARDA (61). Several accessions were donated by Robert Metzger, USDA, Oregon State University, Corvallis (26), 7 were collected by the WGRC, and the remainder (10) from other sources. We also tested 225 CIMMYT wheat lines (Supplemental Table S1) genotyped earlier with GBS SNPs (Gao et al., 2021) thereby inferring the genetic relationships between A-genome diploids and the hexaploid wheat.

### Genotypic characterization

The tested accessions were grown as single plants in the greenhouse and tissue collected in 96-well plates. The tissues were lyophilized for ∼3 days and ground to a fine powder using Retsch mixer mill MM 400. Genomic DNA extraction and GBS library preparation steps were according to (Singh et al., 2019). We had a total of four multiplexed GBS libraries including one for the pilot study. The pilot study GBS library was 384-plex, whereas the other GBS libraries were 288-plex. We sequenced on the Illumina platform with 150 bp pair-end reads (PE150). The information about GBS of 225 CIMMYT lines can be obtained (Gao et al., 2021).

The TASSEL5 GBSv2 pipeline was used for sequence data processing and genotype calling (Glaubitz et al., 2014). Reads were aligned to a *T*. *urartu* pseudomolecule reference (Ling et al., 2018) using bowtie2 alignment (Langmead et al., 2009) and exported to variant call format (VCF). Filtering of the VCF was done for bi-allelic SNPs using the Fisher exact test with a threshold *P*-value < 0.001 as described previously (Poland et al., 2012b) considering that true variants should represent biallelic homozygous state for inbred accessions. Genotypes for accessions across all A-genome species were called together, followed by extracting variants segregating within each species. The SNPs were filtered for MAF > 0.01, missing <30%, and heterozygous <10% at the population level unless otherwise stated in the particular analysis method (e.g. different filtration was used for allele matching and identifying identical accessions) (Supplemental Figure S12). The percentage missing per genotype was computed and removed any genotype with missing >55%. The imputation was done only once in a separated VCF file for *T*. *monococcum* (einkorn) group which was used to compute genome-wide F_ST_ statistics for selection signatures (Supplemental Figure S12). The R codes used to process the hapmap file and downstream analysis and all required data files are provided as supplemental documents at Dryad digital repository (doi:10.5061/dryad.9zw3r22f6).

The A-genome diploids and wheat lines were genotyped together calling variants on the A-genome of wheat reference genome of Chinese Spring (iwgsc_refseqv1.0) (Appels et al., 2018). We also filtered these SNPs using aforementioned criteria. The unrooted NJ phylogenetic tree of A-genome diploid and wheat lines was generated for investigating the genetic relationship. We followed approach of Singh et al. (2019) to generate NJ tree from GBS sampled population, where clustering was conducted with default parameters of R packages “dist,” “ape,” and “phyclust.”

### Gene bank curation

A-genome species in the WGRC gene bank were curated to identify misclassified and genetically identical accessions. The misclassified accessions identified based on the genetic properties were compared with accessions in the adjusted class morphologically to assure if they were previously assigned or documented to the wrong class. Furthermore, to confirm the ploidy of the misclassified accessions that were grouped far from the major *T*. *urartu* clade and did not exhibit a closer relationship with any diploid A-genome in genetic tree, chromosome counts were made by staining with 4',6-diamidino-2-phenylindole following the details and methods of Koo et al. (2017).

The genetically identical accessions were identified using pairwise allele matching across homozygous and nonmissing sites. We first analyzed the loci identity proportions distribution at genome-wide scale including every possible pair-wise comparison among accessions within a single species. A threshold for allele matching percentage given discrepancies for sequencing errors was then detected by finding a point that separates the local maxima existing around the prefect identity (100%). The identity matrix and percentage allele matching were computed in R using a custom script as described by Singh et al. (2019). The morphological similarity and the geographical relations of the genetically identical accessions were checked for confirmation. Glume color (level of darkness) was used as a morphological marker for cross-validation to affirm the accessions in a genetically identical set have the same or similar phenotypes. The variation in glume color was rated from completely white (0) to dark black (9).

### Population structure

Population structure of A-genome wheat species was analyzed using fastStructure (Raj et al., 2014). The filtered genotyping file for the entire population was used to generate input data file for the fastStructure extracting only sites with segregating homozygous loci, where the het genotype calls were converted to missing (doi:10.5061/dryad.9zw3r22f6). The fastStructure was initially run at *K* = 2–12 with three replications using “simple” prior where K refers to number of population or model complexity. The value of *K* was considered optimum if no further distinct differentiations occurred in the population (Supplemental Figures S13 and S14). An appropriate number of *K* was also obtained using the fastStructure provided utility tool, chooseK.py. For the optimum number of *K*, the program was run using “logistic” prior at *K* = 2–7 with three replications (Singh et al., 2019). The fastStructure output was graphically visualized using an R package POPHELPER (Francis, 2017). Passport information including the classification based on morphology, and the accessions geographical sites were used to group and reorder the samples in population analysis. Accessions that were identified as misclassified were confirmed through morphological evaluation and reordered to subspecies based on the genotype-based grouping and the curated results were plotted.

Phylogenetic clustering was carried out in R using “dist” function (https://www.rdocumentation.org/packages/amap/versions/0.8-18/topics/Dist) and “ape” (Paradis and Schliep, 2019) and “phyclust” (Chen, 2011) packages as in Singh et al. (2019). The branches of an unrooted NJ tree were first colored using the morphology-based classification, and then according to genotype analysis. The morphology-based coloring was particularly focused in identifying misclassified accessions. A-genome species population genetic structure was also dissected using PCA of genomic data. For PCA, we estimated the eigenvalues and eigenvectors on R using the “e” function in A matrix obtained from the rrBLUP (Endelman, 2011; Singh et al., 2019).

### Analysis of genetic diversity

A-genome species genetic diversity was assessed by computing the Nei’s diversity index (Nei, 1973) using filtered genotyping markers. We computed the Nei’s indices of (1) all A-genome accessions together, (2) each species and subspecies independently, (3) the races within the subspecies, and (4) and the core collections. Number of segregating loci per group was determined (Table 1). The MAF for each species was also plotted to discern the proportions of rare variants in respective populations. A pairwise F_ST_ (Nei, 1987) also was computed between the species and subgroups separated by the population analysis (Singh et al., 2019).

### F_ST_ within einkorn and selection signature

We computed a genome-wide F_ST_ statistic for variants within the einkorn group using R (R Core Team 2019) as described (Porto-Neto et al., 2013). This method computes F_ST_ statistic based on pure drift model (Nicholson et al., 2002). We also compared the output by computing the Cockerham and Weir F_ST_ statistic (Weir and Cockerham, 1984) using VCFtools (Danecek et al., 2011). The *T*. *monococcum* VCF file separated from the original VCF with biallelic variants was further filtered keeping SNPs with MAF > 0.01, missing <30% and heterozygous <10% followed by imputation using Beagle version 5.1 (Browning et al., 2018). The filtered and imputed genotyping information was used to derive the F_ST_ values. To balance the population sizes of domesticated and wild einkorn, we randomly chose 145 wild einkorn accessions to match the number of 145 domesticated accessions. The F_ST_ were plotted using ggplot2 in R (R Core Team 2019) and the raw F_ST_ plots were smoothed using Lowess method (Pintus et al., 2014) to find the genomic regions with extreme F_ST_. To define the selection signal peak, we considered outlier F_ST_ values that were more than three standard deviation (3*σ*) over genome-wide average as the threshold.

### Core collections

Core collections of *T*. *urartu*, and *T*. *monococcum* (wild and domesticated einkorn) were selected taking allelic diversity, genotype coverage, geographical representation, and phenotypic variation (glume color) into consideration. From the genotyping file containing segregated loci (Table 1), heterozygous genotypes were masked before running the core accessions selection software GenoCore (Jeong et al., 2017). We ran GenoCore with the default parameters: -d 0.01% and -cv 99%. The positions of the selected samples within the phylogenetic tree and PCA clusters were observed through coloring the selected core accessions versus all other samples. Also, the geographical representations were evaluated marking the selected versus remaining accessions in the google map using GPS Visualizer (https://www.gpsvisualizer.com). To ensure phenotypic variations in the selected core sets, we considered the glume color score (Supplemental Table S4) as a reference variation. The Nei’s diversity index (1987) of core sets were also computed (Nei, 1987).

## Accession numbers

Raw sequence data obtained from GBS, the fastq files, has been deposited at the National Center for Biotechnology Information (NCBI) SRA database with the BioProject accession PRJNA744683 (https://www.ncbi.nlm.nih.gov/sra/PRJNA744683). The GBS key file with required information for demultiplexing and further detail about the SRA deposited fastq files can be obtained at Dryad digital repository (doi:10.5061/dryad.9zw3r22f6). All data are available in the manuscript or the supplemental resources and at the Dryad digital repositories https://datadryad.org/stash/dataset/doi:10.5061%2Fdryad.9zw3r22f6.

## Supplemental data

The following materials are available in the online version of this article.

**Supplemental Figure S1.** The *T*. *urartu* clade and subsp. *aegilopoides* α race clade in an unrooted NJ tree.

**Supplemental Figure S2.** Threshold determination for declaring identifying genetically identical accessions.

**Supplemental Figure S3.** An unrooted NJ tree of wheat (*T*. *aestivum* L.) and A-genome species: *T*. *urartu*, subsp. *aegilopoides*, and subsp. *monococcum*.

**Supplemental Figure S4.** Chromosome count for ploidy level of a putative misclassified wild wheat accession TA10881.

**Supplemental Figure S5.** PCA plot for A-genome wheat species with two major PCs.

**Supplemental Figure S6.** MAF plots of A-genome diploid species.

**Supplemental Figure S7.** The F_ST_ curve showing selection signal for einkorn wheat on chromosome 3A.

**Supplemental Figure S8.** An unrooted NJ phylogenetic tree of A-genome wheat species showing the accessions in the core collections and all other accessions in respective clades.

**Supplemental Figure S9.** PCA plot of A-genome wheat accessions indicating all versus core accessions.

**Supplemental Figure S10.** Geographic map of A-genome diploid wheat accessions (small markers) and the accessions in core collection (large markers).

**Supplemental Figure S11.** Diagram showing three different taxonomic classification systems of einkorn wheat.

**Supplemental Figure S12.** Flow diagram showing filtering criteria for different subsets of A-genome diploid collection used to generate SNP matrixes.

**Supplemental Figure S13.** Determining optimum value of K for fastStructure analysis of A-genome species.

**Supplemental Figure S14.** Admixture analysis from *K* = 2 to *K* = 12 with corresponding regions of collection noted for each group.

**Supplemental Table S1.** List of A-genome accessions, their origin, and the genetically identical set (separate file).

**Supplemental Table S2.** The misclassified A-genome species accessions, their previous class based on morphology, and the updated class/group based on the genotyping.

**Supplemental Table S3.** Number of accessions with common PI numbers that clustered in corresponding groups in this experiment and a past experiment.

**Supplemental Table S4.** Core collections of A-genome species (separate file).

**Supplemental Table S5.** Pairwise F_ST_ coefficients among the subgroups within α race of subsp. *aegilopoides* (wild einkorn) and the admixture groups.

**Supplemental Text S1.** Coding sequence of gene for *Btr1* in *T*. *monococcum* subsp. *monococcum*.

## Supplementary Material

kiac006_Supplementary_Data

## Notes

J.P. and J.R. designed the experiment and conceptualized the study. J.R., S.W., D.W., and B.E. carried out experiments and data collection, growing of plant materials, and germplasm. D.-H.K. performed the cytological work. L.A. conducted the computational analysis and phenotypic data collection. N.S. assisted in computational work. J.P. and B.F. supervised the experiment. L.A. and J.P. wrote the manuscript. All the authors have read and approved the manuscript for publication.

The author responsible for distribution of materials integral to the findings presented in this article in accordance with the policy described in the Instructions for Authors (https://academic.oup.com/plphys/pages/General-Instructions) is Laxman Adhikari (laxman.adhikari@kaust.edu.sa).
