# Supplementary material for: Genetic characterization and curation of diploid A-genome wheat species
Source: Plant Physiol. 2022 Feb 3;188(4):2101–14. doi: 10.1093/plphys/kiac006 (PMC8968256; doi:10.1093/plphys/kiac006)
Supplement: kiac006_Supplementary_Data [file kiac006_supplementary_data.zip › SupplementaryDoc.Manuscript-Agenome-Adhikari_02112022-R2.pdf]

Supplemental Data

Genetic Characterization and Curation of Diploid A-Genome Wheat Species

Laxman Adhikari[1,2], John Raupp[1], Shuangye Wu[1], Duane Wilson[1], Byron Evers[1], Dal-Hoe Koo[1], Narinder Singh[1,‡], Bernd Friebe[1] and Jesse Poland[1,2,*]

[1] Department of Plant Pathology, Kansas State University, Manhattan, KS; Wheat Genetic Resource Center (WGRC), Kansas State University, Manhattan, KS 66502, USA

[2] Center for Desert Agriculture, King Abdullah University of Science and Technology (KAUST), Thuwal, 23955-6900, Saudi Arabia

[‡] Current address: Bayer Crop Science, Chesterfield, MO 63017, USA

[*] Corresponding author: jpoland@ksu.edu, jesse.poland@kaust.edu.sa

Supplemental Figures

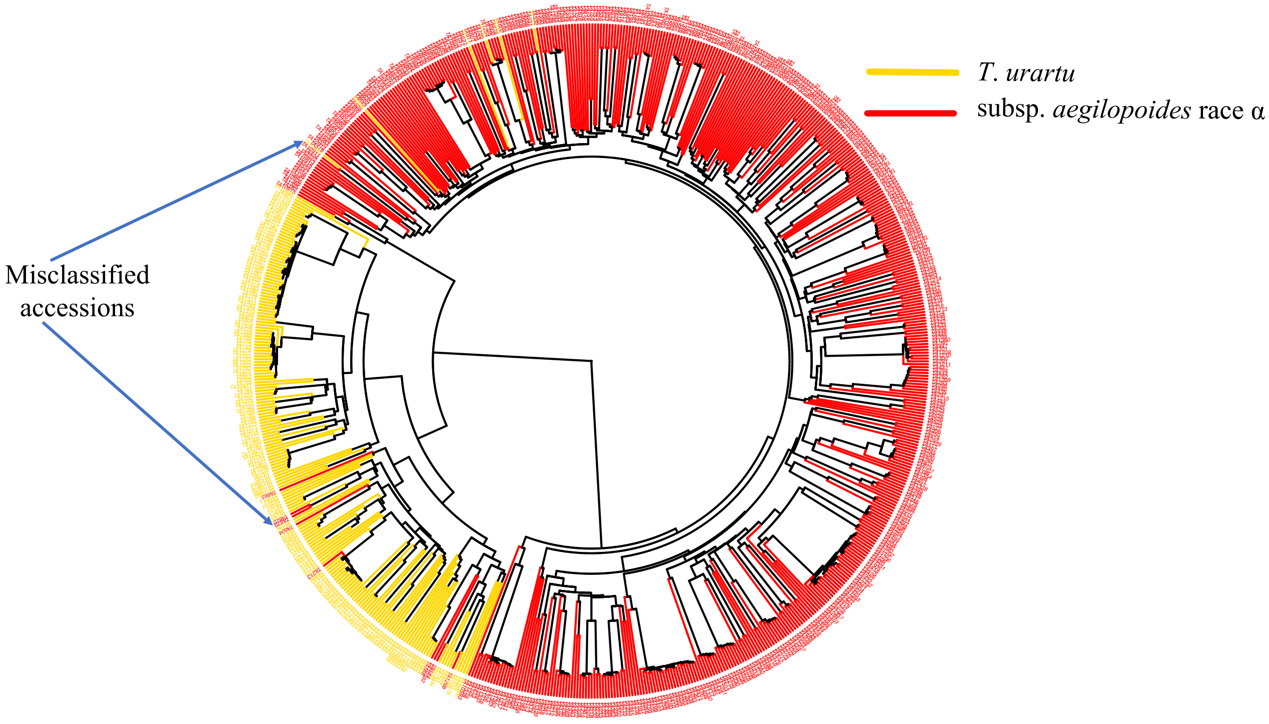

Supplemental Figure S1. The *T. urartu* clade and subsp. *aegilopoides* α race clade in an unrooted Neighbor-Joining (NJ) tree. Denoted with arrows are misclassified accessions between the two groups. The red branches within the gold-colored clade and the gold branches within the red clade reflect the misclassified accessions.

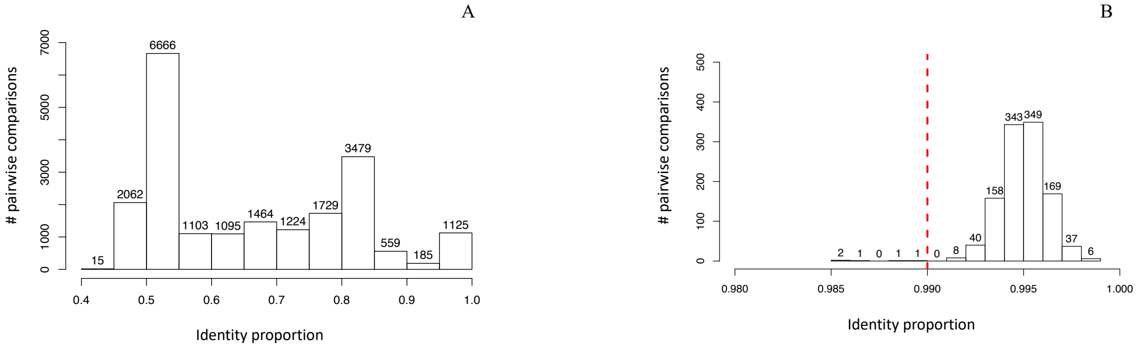

Supplemental Figure S2. Threshold determination for declaring identifying genetically identical accessions. (A) Percentage identity versus the number of comparisons among 196 accessions in *T. urartu* (B) Percentage identity versus the number of pairwise comparisons for the accession pairs in *T. urartu* that had near perfect (≥ 99%) identity.

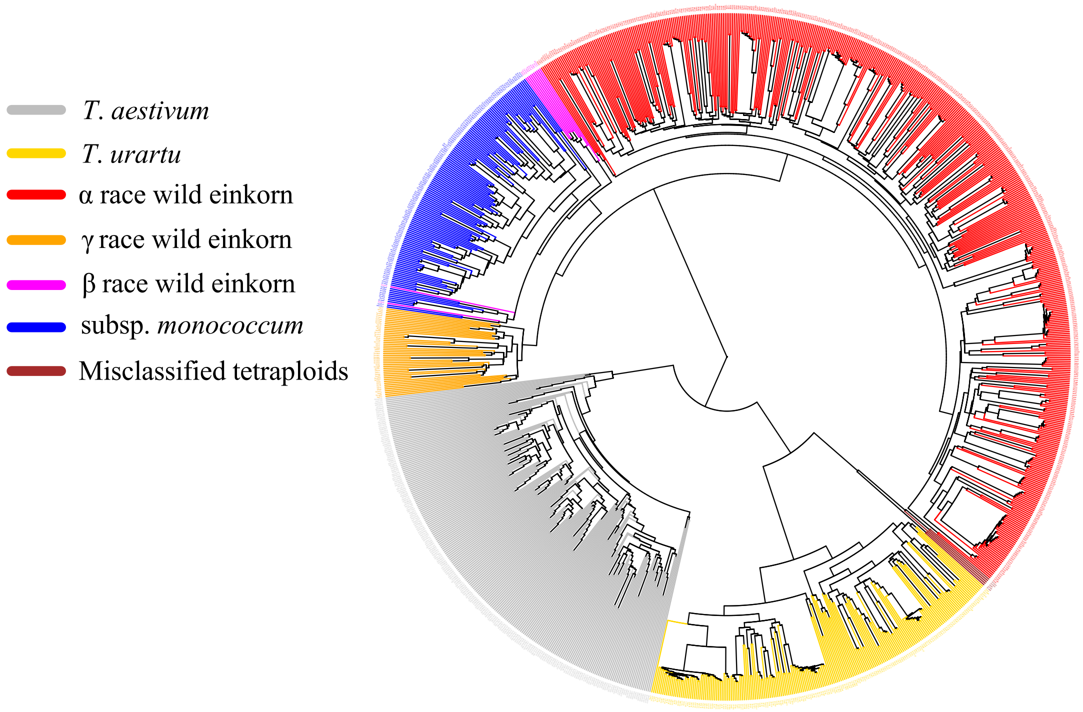

Supplemental Figure S3. An unrooted Neighbor-Joining (NJ) tree of wheat (*T. aestivum* L.) and A-genome species: *T. urartu*, subsp. *aegilopoides*, and subsp. *monococcum*. The tree branches are colored based on the genetic grouping of the accessions after correcting misclassified accessions. Wheat (gray), *T. urartu* (yellow), domesticated einkorn (blue), wild einkorn race α (red), wild einkorn race γ (orange), and wild race β (magenta) and misclassified tetraploids (brown) are shown.

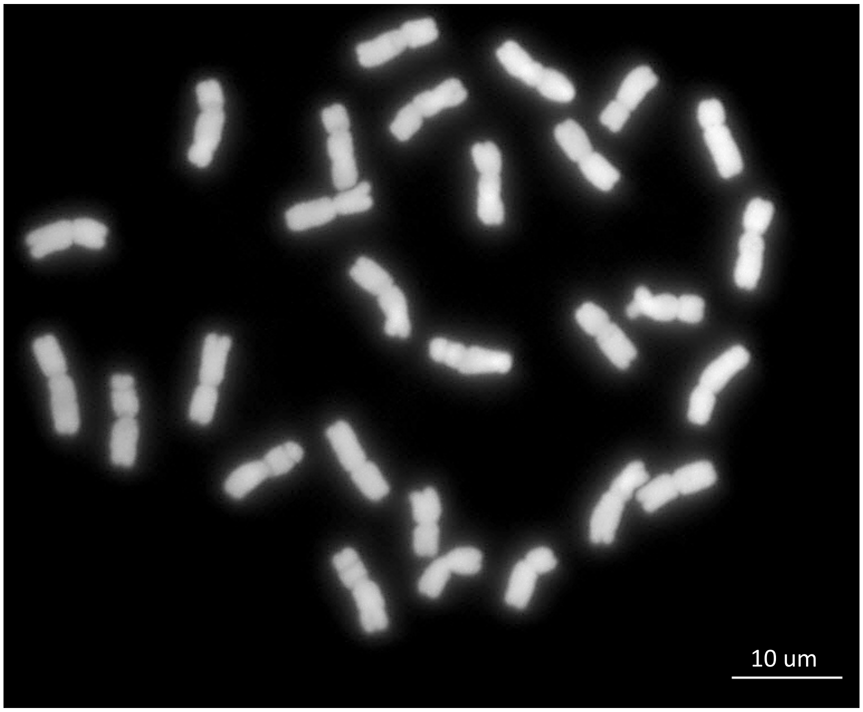

Supplemental Figure S4. Chromosome count for ploidy level of a putative misclassified wild wheat accession TA10881. Prior to genotyping and cytological confirmation, the accession was incorrectly grouped under *T. urartu*. Chromosomes from mitotic metaphase cells were stained with 4',6-diamidino-2-phenylindole (DAPI) which confirmed the accession as tetraploid (2n=4x=28) and thus verified the genotyping-by-sequencing (GBS) based genetic grouping.

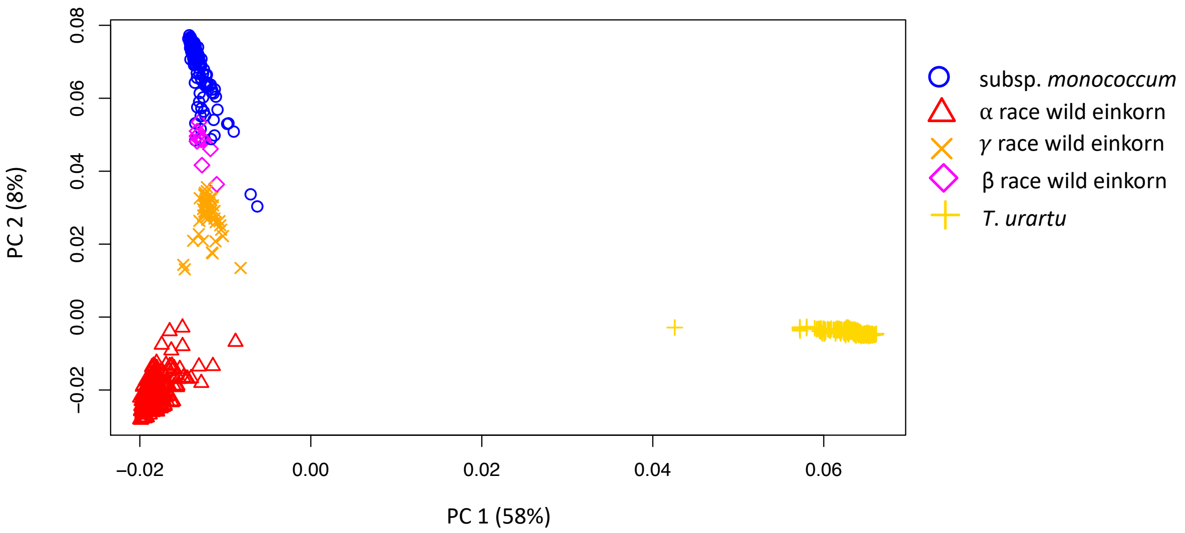

Supplemental Figure S5. Principle component analysis (PCA) plot for A-genome wheat species with two major PCs. The three races α, γ and β within the wild einkorn group are denoted accordingly.

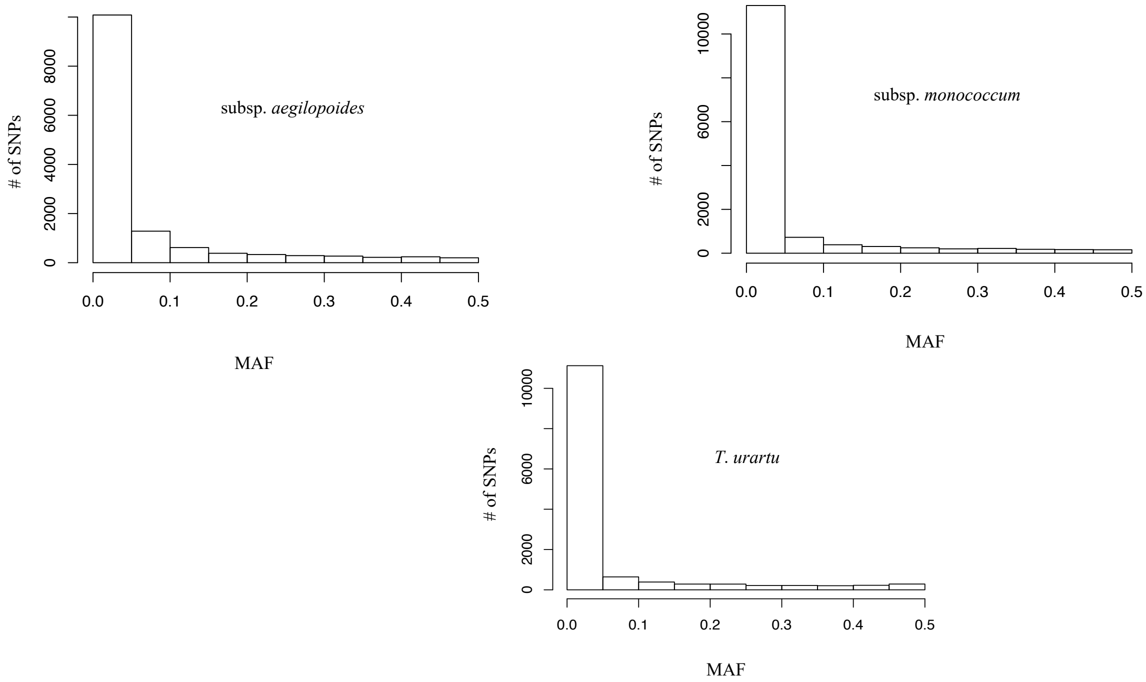

Supplemental Figure S6. Minor allele frequency (MAF) plots of A-genome diploid species. There are three panels indicating: (**a**) *T. monococcum* subsp. a*egilopoides*, (**b**) *T. monococcum* subsp. *monococcum*, and (**c**) *T. urartu.* The Y-axis of each panel has number of single nucleotide polymorphisms (SNPs).

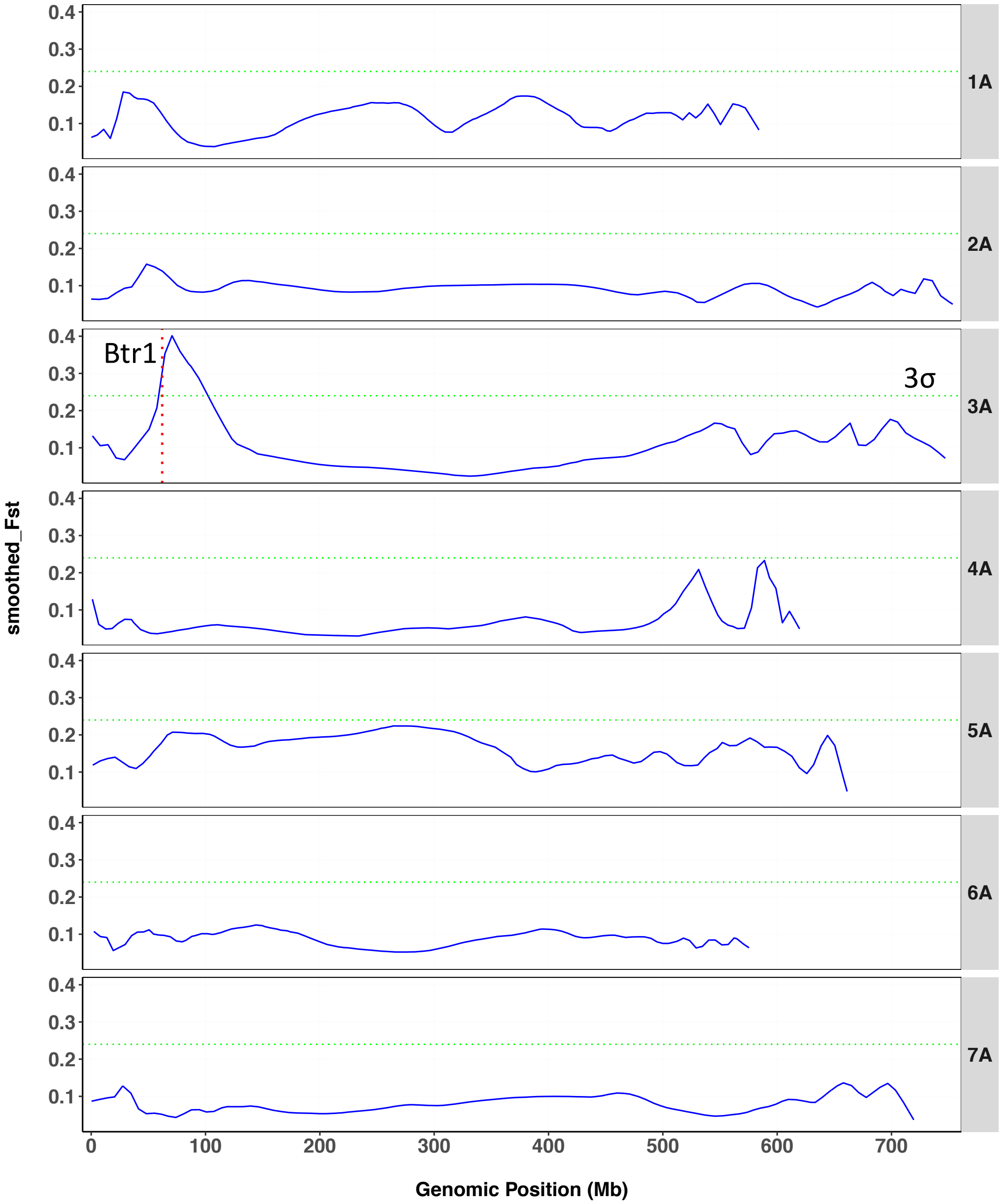

Supplemental Figure S7. The fixation index (F_{ST}) curve showing selection signal for einkorn wheat on chromosome 3A. The strongest signal was located at 60-90 Mb after smoothing the curve. The horizontal green line indicates the threshold (0.24) at 3σ above the mean. The red vertical line at 62 Mb on chromosome 3A indicates the location of non-brittle rachis 1 (*Btr1*).

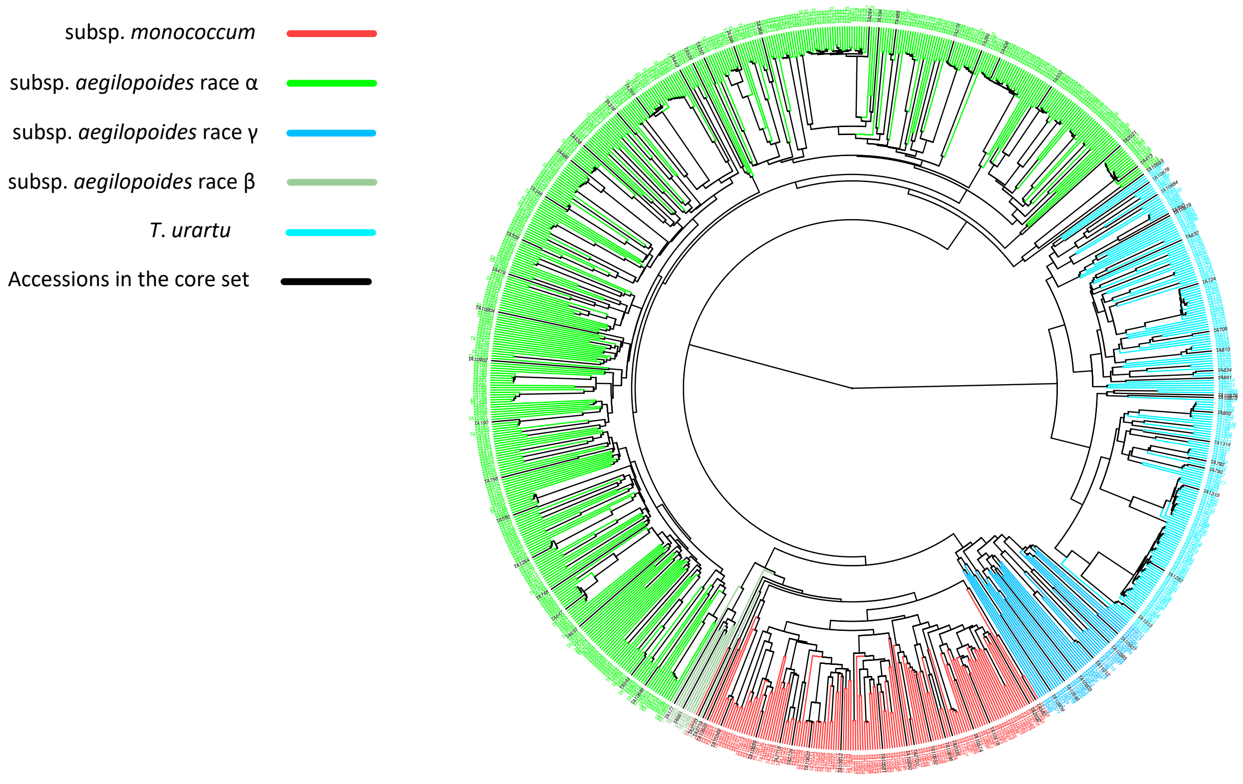

Supplemental Figure S8. An unrooted Neighbor-Joining (NJ) phylogenetic tree of A-genome wheat species showing the accessions in the core collections and all other accessions in respective clades. Black branch reflects the accessions in the core collection, and the golden branch indicates all other accessions that are not in the core collections.

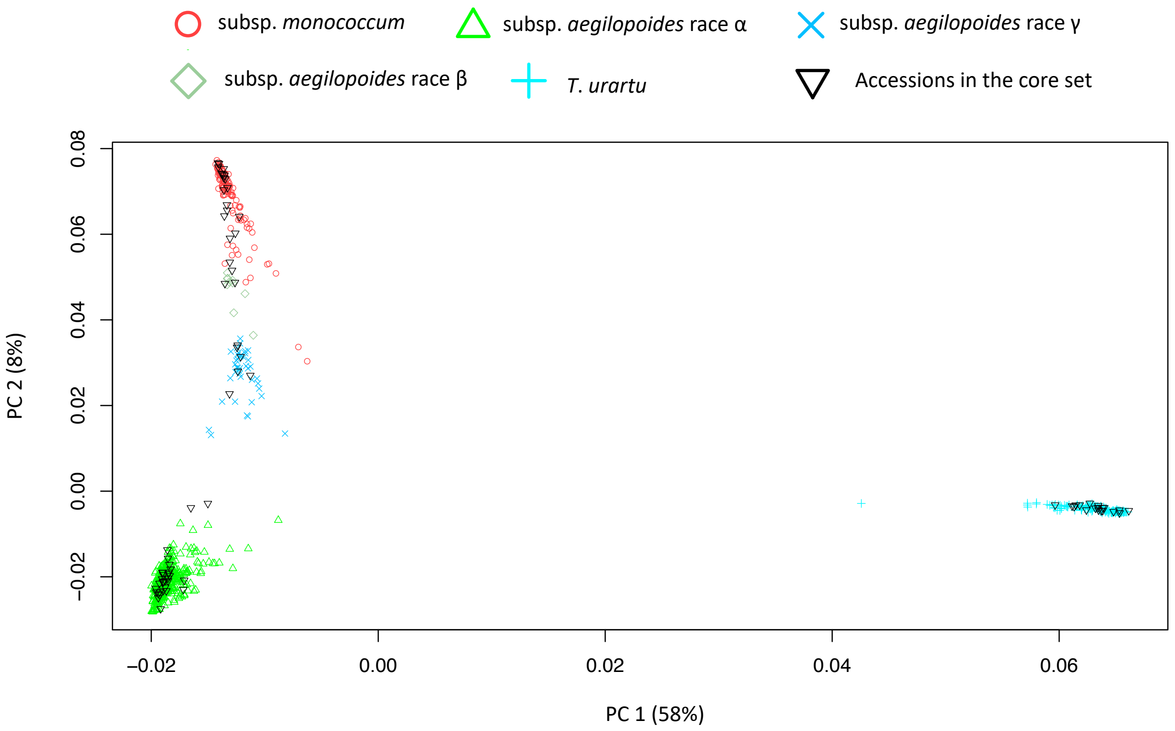

Supplemental Figure S9. Principle component analysis (PCA) plot of A-genome wheat accessions indicating all versus core accessions. The PCA plot showed partitioning of diverse groups within the species and the accessions selected in the genetic cores (black triangles).

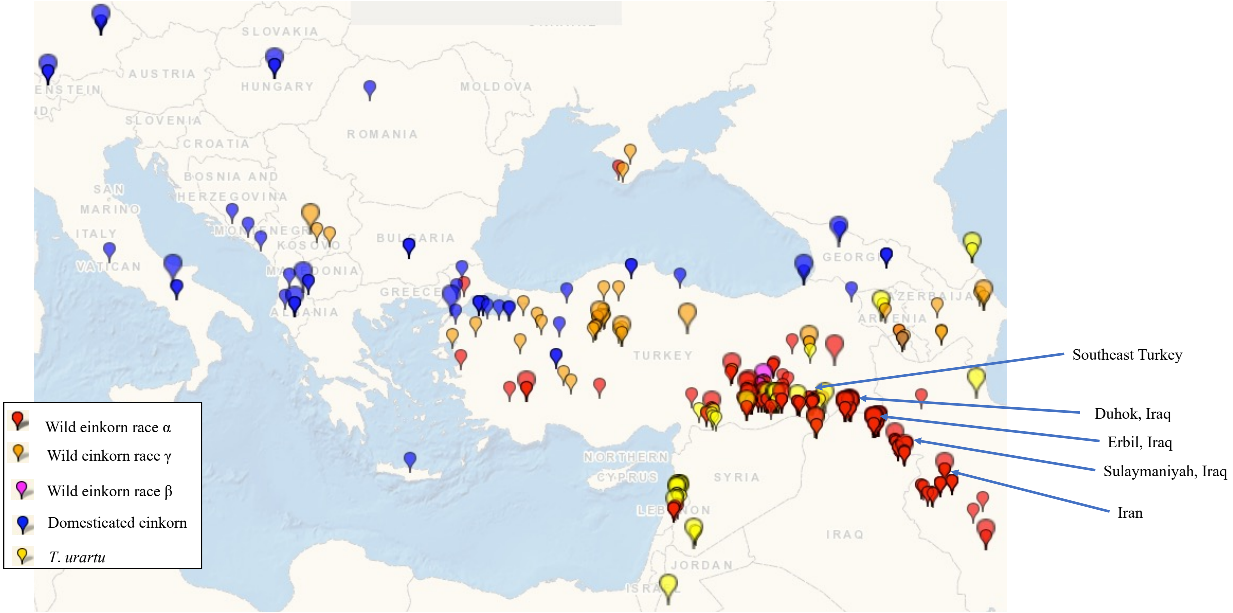

Supplemental Figure S10. Geographic map of A-genome diploid wheat accessions (small markers) and the accessions in core collection (large markers).

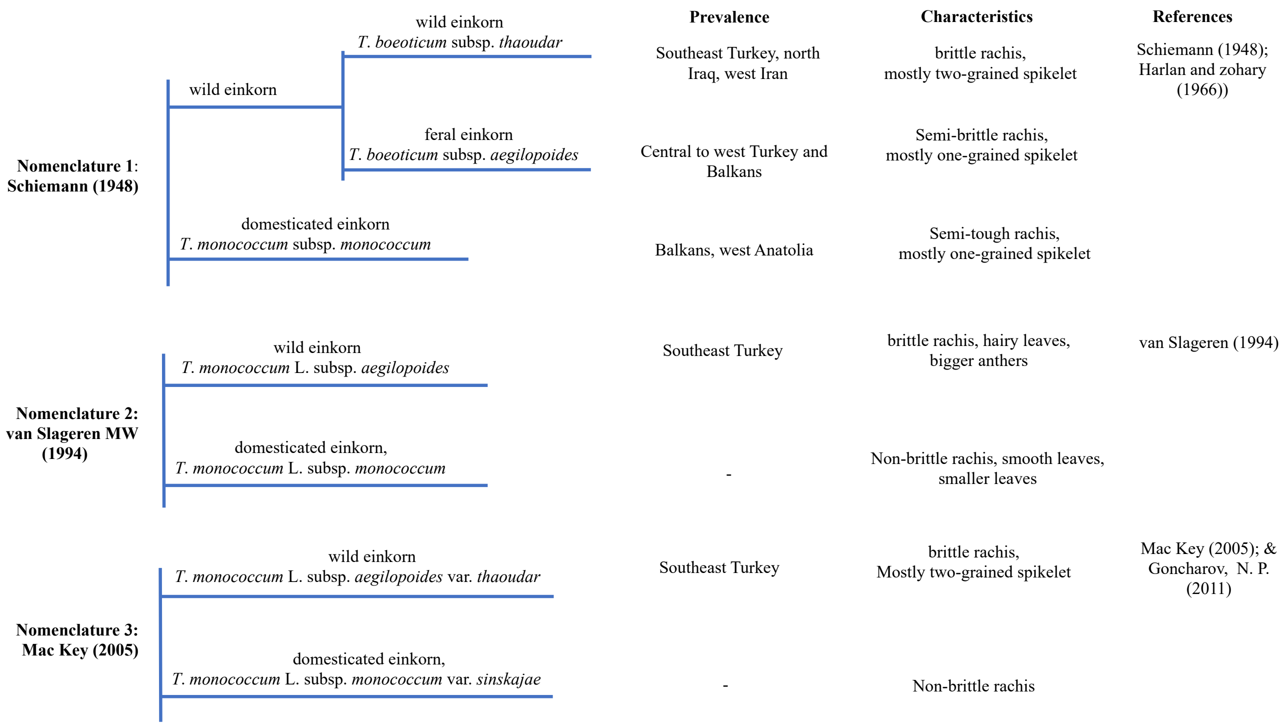

Supplemental Figure S11. Diagram showing three different taxonomic classification systems of einkorn wheat. In the present study we follow the taxonomic classification system of van Slageren (1994).

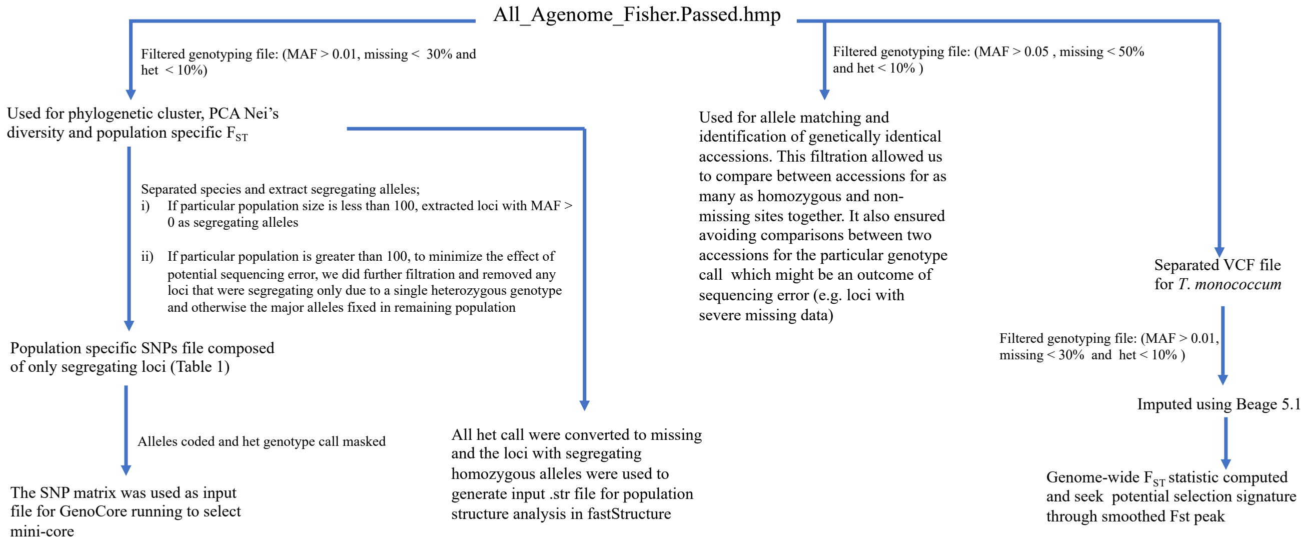

Supplemental Figure S12. Flow diagram showing filtering criteria for different subsets of A-genome diploid collection used to generate single nucleotide polymorphism (SNP) matrixes. The minor allele frequency (MAF), heterozygous (het) loci and missing data were filtered with the variant call format (VCF) file as shown in the diagram. The principal component analysis (PCA) was conducted for population structure and ancestry analysis.

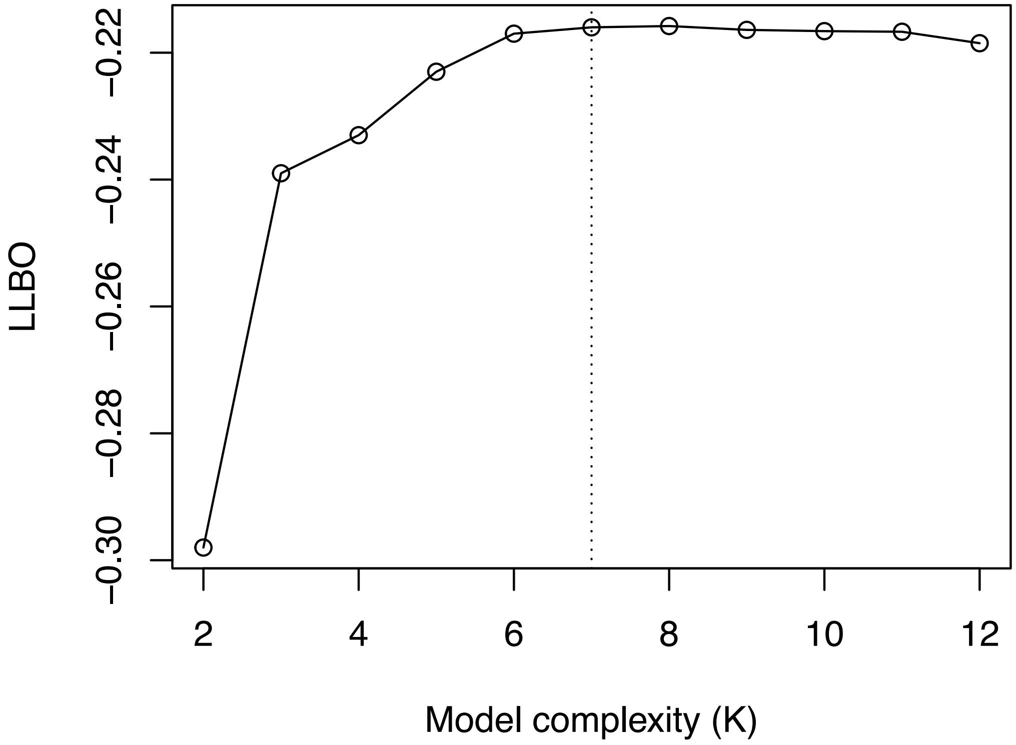

Supplemental Figure S13. Determining optimum value of K for fastStructure analysis of A-genome species. Optimum value of K (=7) was indicated using dashed line and was identified by fastStructure (with the simple prior). The Y-axis represents the log-marginal likelihood lower bound (LLBO), and the X-axis has different values of K.

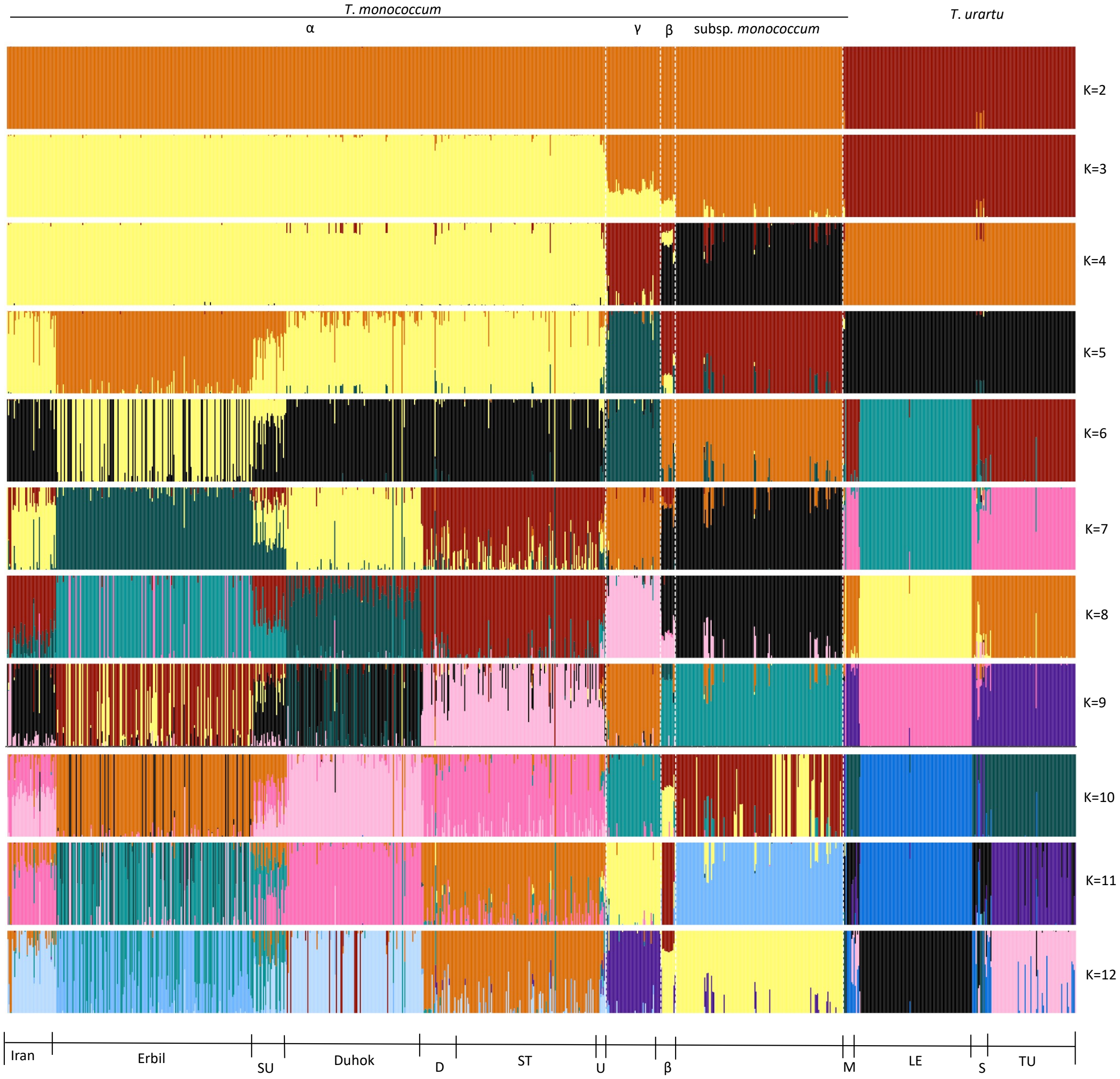

Supplemental Figure S14. Admixture analysis from K=2 to K=12 with corresponding regions of collection noted for each group. We did not see any further differentiation of population beyond K=7. Different abbreviations were given to represent the accessions collection points: Southeast Turkey (ST), Sulaymaniyah (SU) Iraq, random different sites (D), unknown sites (U), Lebanon (LE), entire Turkey (TU), Syria (S) and mixed sites (M).

Supplemental Tables

Supplemental Table S1. List of A-genome accessions, their origin, and the genetically identical set (separate file).

Supplemental Table S2. The misclassified A-genome species accessions, their previous class based on morphology, and the updated class/group based on the genotyping.

Accessions	Origin [City, Country]	Previous class	Updated class
TA568	Elazig, Turkey	subsp. *aegilopoides*	*T. urartu*
TA604	Mardin, Turkey	subsp. *aegilopoides*	*T. urartu*
TA622	Sanliurfa, Turkey	subsp. *aegilopoides*	*T. urartu*
TA665	Sanliurfa, Turkey	subsp. *aegilopoides*	*T. urartu*
TA670	Sanliurfa, Turkey	subsp. *aegilopoides*	*T. urartu*
TA684	Sanliurfa, Turkey	subsp. *aegilopoides*	*T. urartu*
TA1268	Sirnak, Turkey	subsp. *aegilopoides*	*T. urartu*
TA10592	Mus, Turkey	subsp. *aegilopoides*	*T. urartu*
TA213	Arbil, Iraq	subsp. *aegilopoides*	*T. urartu*
TA1325	Diyarbakir, Turkey	subsp. *aegilopoides*	Tetraploid
TA1369	Arbil, Iraq	subsp. *aegilopoides*	Tetraploid
TA10915	As Suwayda, Syria	subsp. *aegilopoides*	Tetraploid
TA282	Arbil, Iraq	subsp. *aegilopoides*	Tetraploid
TA10881	As Suwayda, Syria	*T. urartu*	Tetraploid
TA717	Mardin, Turkey	*T. urartu*	subsp. *aegilopoides*
TA721	Mardin, Turkey	*T. urartu*	subsp. *aegilopoides*
TA722	Mardin, Turkey	*T. urartu*	subsp. *aegilopoides*
TA727	Mardin, Turkey	*T. urartu*	subsp. *aegilopoides*
TA826	Sanliurfa, Turkey	*T. urartu*	subsp. *aegilopoides*
TA10888	Gaziantep, Turkey	*T. urartu*	subsp. *aegilopoides*
TA10548	¥	subsp. *aegilopoides*	subsp. *monococcum*
TA580	¥	subsp. *aegilopoides*	subsp. *monococcum*

¥ = Originally obtained from Department of Agricultural Botany; University of Reading; GBR

Supplemental Table S3. Number of accessions with common PI numbers that clustered in corresponding groups in this experiment and a past experiment. Both studies tested only a portion of germplasms from USDA. The α, β, and γ races indicate the three genetic clusters within the wild einkorn as designated in the past study. Past study also grouped these accessions in the same group that we observed, however, they did not discuss the misclassification issue and just listed the accessions based on morphological classification. The * indicates the accessions that we detected as misclassified and need adjustment of class. Four accessions that Kilian et al. (2007) listed as *T. urartu*, but were genetically identified as α race wild einkorn included: TA554 [PI 428000], TA826 [PI 428251], TA879 [PI 427328] and TA2004 [PI 554498]. TA826 and TA2004 were both collected in Sanliurfa Turkey whereas TA554 were from Beqaa, Lebanon and TA879 from Arbil, Iraq.

Grouping based on a past experiment by Kilian et al. (2007)

		α race	β race	γ race	subsp. *monococcum*	*T. urartu*
GBS SNP based clustering	α race	138	-	2	-	4*
	β race	1	4	-	-	-
	γ race	-	-	10	-	-
	subsp. *monococcum*	-	-	-	14	-
	T. urartu	-	1*	-	-	56

Supplemental Table S4. Core collections of A-genome species (separate file).

Supplemental Table S5. Pairwise fixation index (F_{ST}) coefficients among the subgroups within α race of subsp. *aegilopoides* (wild einkorn) and the admixture groups. There were three subgroups (Turkey, Duhok, Erbil) and two admixture groups (Iran and Sulaymaniyah (Iraq)).

	Duhok	Turkey	Iran	Sulaymaniyah
Erbil	0.21	0.22	0.23	0.17
Duhok	-	0.12	0.12	0.15
Turkey	-	-	0.11	0.15
Iran	-	-	-	0.16

Supplemental text S1. Coding sequence of gene for non-brittle rachis 1 (Btr1) in *T. monococcum* subsp. *monococcum*

>btr1_cult_MG596319.1_Triticummonococcum subsp. *monococcum*
haplotypeTmB1_Hap09BTR1gene, cds
atggcgcagccaccgcaatggaaggcgatgtaccagtatgtggcgatacgggcgcacgacggctgcgcccgcgtcgaggaaagtgtcg
ccgccgcgcgtagggagctggcgtccccgctggtgctggacacccgcaacgctgctgggagttacaccttgttgcattccgcgatgaccc
acgtcgagcacgcgtccggctgcctctccggcgtcatattcagcatgctggtggccgagctcctggcgctccatggctgcggggccgtcc
cgtcgaggccggtggctggcatcggtgacctccgccgcgaccgcgacgaccacgacgagtggctcgctctgagcaggctcgaggcca
ccagggagcaggcccaggacgcgctccgcggggtggagggtaccttcaccctcctggcctccgtccggttcatgcttcacagccggacc
gccgacgctgccgggcgccggcaagtcatggaagagcagctccacgccgccgccgtcgaactccaggccgtggtgggcagcgtggcc
aacatgtccgcgctggccttcttggccacccagcctgccatccgcaaccgcatccagtga

References:

Goncharov, N.P. Genus Triticum L. taxonomy: the present and the future. Plant Syst Evol 295, 1–11 (2011). https://doi.org/10.1007/s00606-011-0480-9

Harlan, J. R., & Zohary, D. (1966). Distribution of Wild Wheats and Barley. Science, 153(3740), 1074-1080. https://doi.org/10.1126/science.153.3740.1074

Kilian, B., Özkan, H., Walther, A., Kohl, J., Dagan, T., Salamini, F., & Martin, W. (2007). Molecular Diversity at 18 Loci in 321 Wild and 92 Domesticate Lines Reveal No Reduction of Nucleotide Diversity during Triticum monococcum (Einkorn) Domestication: Implications for the Origin of Agriculture. *Molecular Biology and Evolution*, *24*(12), 2657-2668.

Mac Key J (2005) Wheat: its concept. evolution and taxonomy. In:Royo C et al. (eds) Durum wheat breeding. Current approaches and future strategies, vol. 1. CRC Press, Boca Raton, pp 3–61.

Schiemann, E. (1948). Weizen. *Roggen, Gerste: Systematik Geschichte und Verwendung, Verlag Gustav Fischer, Jena, Germany*.

van Slageren, M. W. (1994). Wild wheats: a monograph of Aegilops L. and Amblyopyrum (Jaub. & Spach) Eig (Poaceae). *Wageningen Agricultural University Papers*(94-7). Wageningen & ICARDA, Aleppo
